# Inactivation of PETase at Interfaces Inhibits PET Plastic Depolymerization

**DOI:** 10.1021/acssuschemeng.5c13215

**Published:** 2026-05-20

**Authors:** Alecia Robinson, Hannah Lippincott, Chris E. MacFarlane, David J. Kelaita, Catherine Moul, Gur Pines, Daniel K. Schwartz, Joel L. Kaar, Jason T. Boock, Jason A. Berberich

**Affiliations:** † Department of Chemical, Paper and Biomedical Engineering, 6403 Miami University, Oxford, Ohio 45056, United States; ‡ Department of Chemistry and Biochemistry, Miami University, Oxford, Ohio 45056, United States; § Department of Chemical and Biological Engineering, University of Colorado, Boulder, Colorado 80309, United States; ∥ Institute of Plant Protection, Agricultural Research Organization—Volcani Institute, Rishon LeZion 7505101, Israel

**Keywords:** PETase, PET, biocatalysis, air−liquid interface, plastics recycling, interfacial inactivation, enzyme stability

## Abstract

Enzymatic depolymerization of polyethylene terephthalate (PET) is a promising alternative to traditional recycling showing promise in addressing issues with high energy demands and challenges associated with mixed-waste streams. While engineered, thermostable PETase variants exist, achieving high yields under industrially relevant process conditions remains a significant hurdle. Poor thermostability and PET crystallinity are often blamed for low conversions, but these factors do not fully account for the low productivity observed in all cases. This suggests that other poorly understood phenomena may be limiting the catalytic efficiency of PETase warranting further investigation. This work systematically explores the impact of mixing, and in particular, the presence of the air–water and the solid–liquid interfaces, on PETase stability. We found that PET degradation plateaus while soluble PETase activity is lost at increasing mixing rates when an air–water interface is present. It is hypothesized that the enzyme adsorbs to the air–water interface leading to denaturation, aggregation and precipitation. Additional studies demonstrate that the hydrophobic PET film negatively impacts enzyme stability and the rate of enzyme inactivation is also proportional to the PET surface area. Traditional approaches to mitigate enzyme inactivation at interfaces, such as using blocking agents, were effective in preserving enzyme activity, but resulted in significantly reduced PET degradation rates. In contrast, PEGylation of PETase yielded improved stability and enhanced PET degradation during mixing. These findings help explain the significant conversion discrepancies reported in previous studies and demonstrate a robust strategy for improving PETase performance under industrially relevant conditions.

## Graphical abstract

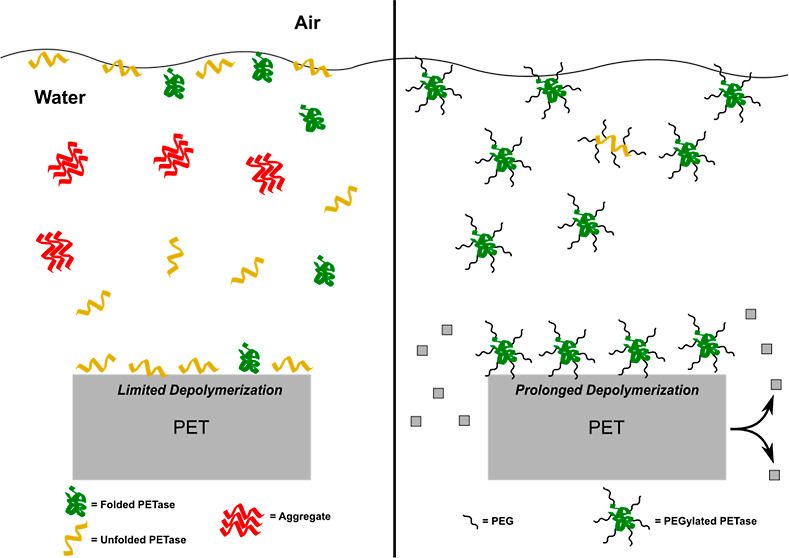

## Introduction

Due to its low cost and versatility, the synthetic polymer poly­(ethylene terephthalate) (PET) is widely used in food and beverage packaging and textiles. Because of its widespread use, PET composes a significant portion of the 8300 megatonnes of plastic produced since the 1950s and approximately 10% of the almost 400 megatonnes per year of plastic that is produced currently.^1–3^ Due to the considerable environmental and potential health impacts of plastics and microplastics, the need for recycling is more important than ever.^4–6^ However, the development of an economically viable chemical recycling approach is difficult due to high energy requirements and the challenges associated with separation and sorting of the incoming mixed plastic recycling streams.^3,7–9^ To address these challenges, there has been growing interest and modest success in using enzymes to hydrolytically degrade PET. The ability of enzymes to degrade PET at lower temperatures reduces energy requirements. In addition, their high selectivity limits the formation of side products, which simplifies monomer separation, facilitates repolymerization, and creates an opportunity for a circular plastic economy.^3,10–16^

A number of enzymes have been identified that are capable of degrading PET to varying degrees, including lipases,^17^ cutinases^18–20^ and the recently discovered PETases.^21–23^ Currently, significant effort is focused on engineering these enzymes using a variety of approaches to enhance their activity on PET as well as their stability at higher temperatures.^24^ The latter is desirable because the use of higher temperatures increase the flexibility of the polymer chains in the amorphous regions of PET, making them more accessible to enzymatic degradation.^25–30^ Despite improvements in PETase thermostability, significant PET conversions at high solid loadings at scale have remained challenging.^12,31,32^

The low thermostability^28,33,34^ and poor performance on crystalline PET^35–38^ are two primary factors typically attributed to limiting the productivity and conversion of PETase-mediated degradation. A recent paper comparing four PET degrading enzymes (LCC^ICCG^, FAST-PETase, HotPETase, and PES-H1^L92F/Q94Y^) found that PET degradation under identical conditions was largely temperature and enzyme dependent.^12^ They observed that enzyme performance did not necessarily correlate with the thermostability of the enzyme (based on *T*_m_), with HotPETase performing significantly worse than PES-H1^L92F/Q94Y^ (26.4% conversion vs 90.1%) at PET loadings of 16.5% (w/w), even though PES-H1^L92F/Q94Y^ has a lower *T*_m_ (77.6 °C vs 80.7 °C).^12^ Interestingly, they also found that the performance of PES-H1^L92F/Q94Y^ did not translate across different reactor volumes and solid loadings. They observed significantly higher conversions at larger volumes and higher PET loadings than for small volumes with lower PET loadings.^12^ As noted by the authors, the differences in performance are presumably complex and likely involve other phenomenon besides low stability and product inhibition, the latter of which has also been reported previously.^34,39,40^ Other explanations of poor PET degradation performance across studies have included unproductive binding,^41^ surface crowding^33,42^ and mixing.^34,43^

Despite the plethora of prior studies, a mechanistic understanding of the inactivation of PETases remains incomplete. Comparison of enzyme activities, stabilities, and extents of PET degradation across studies is difficult due to differences in reaction temperatures, pH, and buffers. Furthermore, conditions used in studies are often selected to yield the highest conversion on small scales; however, these conditions are not necessarily optimal or suitable for large scale applications, such as omitting mixing which would result in mass transfer issues. For example, the addition of DMSO and the use of nonmixed conditions was found to increase the extent of PET product formation for *Is*PETase and its more thermostable variants.^28,34^ FAST-PETase was shown to be effective in rapidly degrading various forms of PET, but none of the reactions were noted to be performed in the presence of mixing, and fresh enzyme was periodically added to increase conversions.^25^ The inclusion of additives such as ionic surfactants^44,45^ and PEG^46^ have also been demonstrated to improve PET degradation rates and enzyme stability. Similarly, HotPETase, a more thermostable *Is*PETase variant, was shown to be effective at degrading PET at elevated temperatures, but all reactions were performed with 4% Bug Buster, which is composed of a proprietary mixture of nonionic and zwitterionic detergents.^26,47^ This wide range of reaction conditions suggests that a variety of factors impact the extent of the enzymatic degradation in addition to the *T*_m_ with the addition of different chemical additives improving degradation in some cases.

A critical, yet largely overlooked aspect of PET degradation is the impact of interfaces on enzyme stability and thus performance. While a few studies have noted the negative effects of mixing, these phenomena have not been rigorously investigated.^34,43^ It is well-known that interactions with interfaces can initiate protein unfolding, resulting in protein aggregation,^48–54^ especially with highly surface-active proteins. Moreover, interface-mediated protein unfolding and aggregation is often exacerbated by agitation (e.g., mixing), which results in alternating growth and reduction cycles of liquid–vapor interfacial area.^52,55–59^ In this work, we systematically explored the distinct roles of both the air–liquid and the PET-liquid interfaces on PETase activity. By carefully controlling the reaction conditions, we were able to deconvolute their individual contributions to enzyme inactivation. Our results specifically showed that wild-type *Is*PETase and its engineered variants are unstable at the air–water interface created during mixing and that the solid–liquid interface created with the PET film also contributes significantly to PETase inactivation. After exploring common stabilization methods, we demonstrate that modifying PETase with polyethylene glycol (PEGylation) is a highly effective strategy, enabling both improved PETase stability and enhanced PET degradation in the presence of mixing. These findings provide crucial insight that helps to explain the significant discrepancies in conversions reported across previous studies. Furthermore, the learnings from this work may enable the development of rational strategies to improve PETase performance beyond current methods.

## Materials and Methods

Amorphous PET film (ES301445, 2% crystallinity, *T*_g_—77 °C, 0.25 mm thickness, 6 mm diameter and average weight of 9.6 mg) and PET microparticles (ES306031, average particle size >100 μm, 37.7% crystallinity and *T*_g_—65 °C) were purchased from Goodfellow Advanced Materials and were previously characterized.^34,41^ Sodium phosphate, terephthalic acid (TPA), *p*-nitrophenyl acetate (pNA), Triton X-100, Brij 35, Pluronic F127, bovine serum albumin and gelatin Type A from porcine skin were purchased from Sigma-Aldrich. Seal-Rite microcentrifuge tubes, 1.5 mL, were purchased from USA Scientific (Florida). The methoxy polyethylene glycol 5000 *N*-hydroxysuccinimide ester reagent (mPEG-GAS) was from Creative PEGWorks (PSB-2083, MW 5 kDa). All lipids, including 1,2-dioleoyl-*sn*-glycero-3-phospho-(1′-rac-glycerol) (DOPG), 1,2-dioleoyl-*sn*-glycero-3-phosphocholine (DOPC), 1,2-dioleoyl-*sn*-glycero-3-ethylphosphocholine (DOEPC), and 1,2-dioleoyl-*sn*-glycero-3-[(*N*-(5-amino-1-carboxypentyl)­iminodiacetic acid)­succinyl] (nickel salt) (DGS-NTA­(Ni)), were purchased from Avanti Polar Lipids. All solutions were prepared with deionized water from a Milli-Q Millipore system, 18 MΩ.

### Protein Expression and Purification

*Is*PETase, Fast-PETase and HotPETase were expressed in *Escherichia coli* and were purified using immobilized metal affinity chromatography (IMAC) followed by desalting into storage buffer as previously described.^60^ Enzyme purification was verified by SDS-PAGE and total protein concentration determined using a Pierce Micro BCA Protein Assay kit (Thermo Fisher). Purified samples were stored at 4 °C until use. Specific activities of the enzymes were previously characterized using *p*-nitrophenyl acetate and were consistent with reported literature values.^60^

### Circular Dichroism

Far UV circular dichroism (CD) scans of the PETase variants were performed using an Aviv Biomedical Model 435 CD spectrophotometer. PETase samples were prepared at 0.03 mg/mL in 10 mM sodium phosphate buffer (pH 8). The melting temperature was determined by heating the protein sample at a rate of 1.4 °C from 25 to 89 °C. Ellipticity was measured at 220 nm every 2 °C after holding the sample for 0.5 min with an averaging time of 3 s prior to going to the next temperature. The resulting melting curve was fit using a thermodynamic model to determine the melting temperature.^61^

### PET Degradation Activity

Degradation of PET was studied using amorphous PET films and PET microparticles. *Is*PETase and FAST-PETase were prepared in 100 mM sodium phosphate buffer (pH 8.0) while HotPETase was prepared in 50 mM glycine buffer (pH 9.2). Reactions were typically conducted in 1.5 mL Seal-Rite polypropylene microcentrifuge tubes at 35 °C for *Is*PETase, 45 °C for FAST-PETase and 60 °C for HotPETase. The vials were either completely or partially filled as indicated. For reactions with partially filled vials, enzyme solutions (1 mL) were mixed using an Eppendorf Thermomixer C. To understand the impact of tube material on enzyme stability, select experiments were also performed using 2.0 mL glass HPLC vials (Thermo Fisher).

The degradation of insoluble PET results in the release of soluble TPA, mono­(2-hydroxyethyl) terephthalate (MHET) and bis­(2-hydroxyethyl) terephthalate (BHET) and some oligomers. Product formation was monitored by measuring absorbance of the liquid phase at 242 nm in a Biotek Take-3 microvolume plate (path length 0.05 cm) using a Biotek microplate reader as described previously.^12,28^ For samples containing dimethyl sulfoxide (DMSO), absorbance was measured at 260 nm instead due to interference. The estimated concentration of TPA_eq_ produced, which corresponds to the sum of the soluble hydrolysis products, was determined using the molar absorptivity of TPA. The molar absorptivity of TPA in sodium phosphate buffer (100 mM, pH 8.0) was determined to be 12,574 M^–1^ cm^–1^ at 242 nm and 4834 M^–1^ cm^–1^ at 260 nm. Similarly, the molar absorptivity of TPA in glycine buffer (50 mM, pH 9.2) was determined to be 12,822 M^–1^ cm^–1^ at 242 nm and 4944 M^–1^ cm^–1^ at 260 nm. Where necessary samples were diluted in buffer prior to analysis. The percent depolymerization was calculated using the concentration of TPA_eq_ and the theoretical maximum concentration of TPA produced from the complete degradation of the PET added to the reaction. For all experiments, percent depolymerization is reported as the mean ± standard deviation from experiments performed with triplicate samples, and each absorbance was measured in technical duplicate.

### Enzyme Activity Assays

Enzyme activity was monitored using the soluble substrate 4-nitrophenyl acetate (pNA). To each well of a 96 well plate, 10 μL sample was added followed by 180 μL assay buffer (100 mM sodium phosphate buffer, pH 7.0 with 0.1% w/v polyethylene glycol (PEG) 8000). Reaction was initiated by adding 10 μL 200 mM pNA stock solution prepared in 100% acetonitrile (ACN). Change in absorbance was measured at 405 nm at 25 °C for 10 min using a microplate reader (Biotek). To compensate for nonenzymatic hydrolysis of pNA, rates of blank (buffer-only) samples were subtracted from those containing enzyme. Initial rates were calculated from the linear region of the curve. Percent relative activities were determined as the percentage of the rate at each time point relative to the rate at the beginning of the experiment (*t* = 0). Experiments were performed with triplicate samples, and each assay was performed in technical duplicate. Data are presented as the mean value ± the standard deviation.

### Protein Concentration Quantification

Total protein quantification in solution was carried out using a Pierce Micro BCA kit (Thermo Fisher) according to manufacturer specifications and absorbance quantified using a microplate reader at 562 nm. Total protein concentration was determined through comparison to bovine serum albumin standards (2 μg/mL–40 μg/mL).

### Coomassie Stain of Aggregated Protein

Solutions of *Is*PETase (30 μg/mL) were added to 15 mL glass or polypropylene conical tubes and mixed at 1000 rpm on an Eppendorf Thermomixer C. After the soluble protein had precipitated from solution, the buffer was removed from the tubes. Coomassie reagent (Pierce Bradford Protein Assay Kit) was added, and the tubes were placed on a rocker at room temperature for 30 min to stain the protein aggregates.

### Preparation of Small Unilamellar Vesicles (SUVs)

Lipids (DOPG or DOEPC) dissolved in chloroform were mixed in glass vials at the desired ratios, with zwitterionic DOPC used as the balancing lipid. All vesicle formulations contained 5% (w/w) DGS-NTA­(Ni) to facilitate tethering of His-tagged PETase. Chloroform was evaporated under a stream of nitrogen until a dry lipid film remained, which was then rehydrated in 50 mM sodium phosphate buffer (pH 8) to a final lipid concentration of 10 mg/mL. The lipid suspensions were incubated at 42 °C for 30 min to ensure complete hydration and subsequently extruded 21 times through a 50 nm polycarbonate membrane using a mini-extruder (Avanti Polar Lipids) to generate a monodisperse population of small unilamellar vesicles. Enzymes were tethered to the surface of SUVs by mixing at a 1:100 enzyme/tethering lipid ratio and incubating for 1 h at room temperature with gentle rotation.

### PEGylation of FAST-PETase

FAST-PETase was modified by reacting the enzyme with glutaramide succinimidyl estermethoxy-PEG (5000 MW) using a 25:1 NHS-to-primary amines in 100 mM sodium phosphate buffer, pH 8.0 for 12 h. The activity retention of PEGylated PETase was determined by diluting the crude reaction mixture of known enzyme concentration and assaying for activity using pNA. The rate of pNA hydrolysis for PEGylated PETase was subsequently normalized by the rate of pNA hydrolysis for unmodified PETase at the same concentration. To remove nonconjugated PET, the PEG-FAST-PETase was repurified from the crude reaction mixture using a 1 mL His Trap HP (Cytiva) column with a flow rate of 1 mL/min. Single step elution was carried out using elution buffer (50%, 250 mM imidazole). The purification process was verified by SDS-PAGE using protein and iodine stains (for PEG). The protein bands were stained using Coomassie reagent (Pierce) for 1 h at room temperature followed by destaining with Milli-Q deionized water for 90 min. The presence of free PEG in the gel was determined by iodine staining. The Coomassie stained gels were soaked in iodine stain for 1 h followed by destaining with water. The iodine stain consisted of 1.3% (w/v) iodine, 1.0% (w/v) potassium iodide, and 2.5% (w/v) barium chloride in a 0.6 M HCl solution.

## Results and Discussion

### *Is*PETase and Variants are Rapidly Inactivated in the Presence and Absence of PET Films

To understand how the presence of PET contributes to enzyme deactivation, preliminary degradation experiments were performed using *Is*PETase^21^ and the two variants FAST-PETase^25^ and HotPETase^26^ by monitoring both the conversion of PET films as well as remaining soluble enzyme activity. For *Is*PETase, a reaction temperature of 35 °C was selected to match conditions similar to those in Erickson et al.^34^ For FAST-PETase and HotPETase, we chose temperatures (45 °C for FAST and 60 °C for HotPETase) that were just below the optimal temperatures found by Arnal and co-workers.^12^ Studies were performed using amorphous PET films (2% crystallinity) that were mixed at 1000 rpm and soluble PET degradation products were monitored as described previously.^28^ For all enzymes, the progress curves were initially linear but rapidly slowed within 5–10 h while product formation plateaued within 24 h even though significant PET (>93%) was still available. Notably, *Is*PETase showed the least amount (<1%) of PET degradation (Figure S1A).

A number of reasons may explain the decline in PET degradation over time, including inhibition by PET degradation products such as MHET, BHET and TPA^34,40^ and loss of PETase activity. Others have shown that under similar conditions, additions of more enzyme result in further conversion, likely ruling out product inhibition.^26^ The reaction conditions selected were >10 °C below the *T*_m_ of each of the enzymes (48.2 °C for *Is*PETase, 62.8 °C for Fast-PETase and 82.6 °C for HotPETase; Figure S2). To test whether PETase inactivation was the cause of the decline of product formation, samples of the liquid phase were periodically removed from the vials and residual volumetric PETase activity in solution was assayed using pNA. A rapid decline in solution phase enzyme activity for each enzyme under these conditions was observed (Figure S1B). By 3 h greater than 80% of PETase activity was lost and by 24 h the PETases were nearly completely inactivated. The decline in solution phase activity may be explained by deactivation of the enzyme as well as by adsorption of the enzyme to the PET film.^41^ Due to the low concentration of protein in solution, it was difficult to reliably measure the protein concentration after adsorption to the films. Instead, we estimated the protein bound to the PET films using the reported adsorption isotherms for *Is*PETase.^33,41^ Based on the isotherm, we would expect to retain 94% of its initial activity in solution following adsorption of the enzyme to the PET. Adsorption parameters for FAST-PETase and HotPETase are unavailable, but they are likely similar due to the high structural and sequence homogeneity between Hot and FAST-PETase and *Is*PETase.

If the decrease of PET degradation is due to enzyme deactivation in solution and not due to enzyme adsorption to PET, we would expect to observe a decrease in enzyme activity when no PET is present. To test this hypothesis, we ran a similar experiment where the enzymes were mixed at 1000 rpm without the PET films (Figure S1C). Interestingly, for *Is*PETase and the variants, we observed similar rates of inactivation as in the case with PET films. While it is difficult to directly compare these results, this confirms that the apparent inactivation in solution is not completely due to simple adsorption of PETase enzymes to the PET films, since we would expect enzyme activity for extended periods when PET film is present if the enzyme was stable.

### Mixing Leads to Inactivation of *Is*PETase and Variants

Given the apparent inactivation of PETase without PET, we hypothesized that other factors besides surface adsorption may be contributing to the apparent loss of PETase activity in solution. A potential factor that may contribute to inactivation is mixing. While the impact of mixing has been observed previously for *Is*PETase^34^ and DuraPETase,^43^ this impact has not been characterized rigorously. As noted by Arnal and co-workers,^12^ “it is interesting how quickly the PET degradation ceases with mixing, even when the enzyme is incubated 10 to 20 °C below its *T*_m_”. To better understand the impact of mixing in combination with temperature on PETase inactivation, reactions were monitored under a variety of temperatures under mixed and nonmixed conditions. Due to the significantly higher PET degradation observed with FAST and HotPETase in comparison with *Is*PETase, studies focused on these two enzymes.

Consistent with previous findings,^12^ we observed a rapid decline in PET degradation for FAST-PETase at all temperatures studied, including temperatures well below its *T*_m_ under mixed conditions (Figure 1A; Table S1). The PET conversions at short times using FAST-PETase increase with temperature but depolymerization begins to decline within a few hours and plateaus within 24 h independent of temperature when mixed. Interestingly, under nonmixed conditions at 40 and 45 °C, PET degradation slowed only slightly by 48 h. However, at 50 °C, there was little difference between PET degradation under mixed and nonmixed conditions. Similarly, for HotPETase we see a rapid decline in PET depolymerization for all temperatures studied when mixed (Figure 1B), with conversion plateauing by approximately 12 h, slightly faster than observed with FAST-PETase. For HotPETase, higher rates of product degradation were observed initially for all of the nonmixed samples; however, the PET degradation reaction had slowed considerably or stopped for all reaction temperatures by 48 h, regardless of mixing. We observe that as the reaction gets closer to the *T*_m_ of the enzyme, the impact of mixing on the deactivation of FAST and HotPETase during PET degradation becomes smaller. This suggests that while mixing induces the inactivation of PETase at lower temperatures, inactivation at higher temperatures is likely driven primarily by thermal denaturation. For FAST-PETase without mixing, the trade-off between activity and thermal stability was readily apparent. Although slower PET degradation was observed at lower temperatures, higher degrees of depolymerization were observed at longer reaction times due to reduced unfolding at lower temperatures. A similar dependence on temperature for the initial rates and shape of the reaction progress curves was observed for FAST and HotPETase under mixed conditions by Arnal and co-workers.^12^

**Figure 1:**
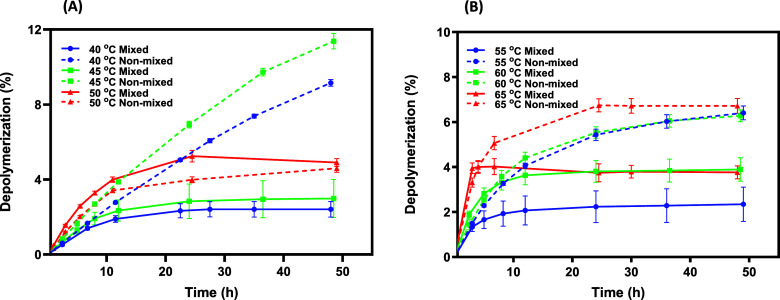
Progress curves for the hydrolysis of amorphous PET using 200 nM FAST-PETase (A) and 200 nM HotPETase (B) under mixed (1000 rpm) and nonmixed conditions. Error bars indicate the standard deviation of experiments performed in triplicate.

The decline in PET degradation rate for all the PETase variants under mixed conditions suggests that the process of mixing plays a critical role in PETase inactivation. To understand this further, we investigated the impact of mixing speed in the absence of PET on the stability of FAST-PETase, which was the most sensitive to mixing. For these experiments, FAST-PETase was mixed at various speeds (0–2000 rpm) in partially filled polypropylene tubes with no PET at 45 °C. Importantly, due to the tubes being partially filled, the presence of headspace created a large and dynamic liquid–air interface during mixing. PETase stability was measured by monitoring the loss of activity via periodically removing samples and assaying enzyme activity. Additionally, enzyme aggregation was monitored by measuring the loss of PETase from solution over time. For these experiments, the concentration of PETase used in these studies was increased to 1 μM to enable accurate measurement of enzyme concentration using the micro BCA assay.

Interestingly, as the mixing rate is increased, the rate of loss of soluble enzyme activity in solution increased (Figure 2A). Although FAST-PETase lost less than 25% of its initial activity when mixed at 0 and 500 rpm over 4 days, the enzyme was much less stable at higher mixing speeds. When mixed at 1000 rpm, the enzyme was inactivated within ∼3 days, with less than 50% of the activity at 24 h. At 1500 and 2000 rpm, complete inactivation was observed after only 12 h. Moreover, the loss of activity in solution correlated with the decline of soluble enzyme (Figure 2B). This was corroborated by the appearance of insoluble enzyme aggregates in solution and adsorbed to the tube walls, which was further verified via staining with Coomassie (Figure S3). The loss of enzyme activity with time was also monitored for FAST-PETase at 200 nM, which matches our earlier reaction conditions. At this lower enzyme concentration, we observed that inactivation was faster, with total loss of activity at high mixing rates within 2 h (Figure S4). More activity loss was found for the 200 nM samples at low mixing rates than we saw for the higher concentration samples, which is not surprising since under dilute conditions a larger fraction of the enzyme may be lost due to interfacial adsorption. While not explored here, the buffer composition, concentration and pH would likely impact PETase stability, especially as the pH nears the isoelectric point of the protein.^62,63^ Under the conditions studied, the inactivation of FAST-PETase led to the formation of insoluble aggregates, which do not appear to reversibly dissolve.

**Figure 2:**
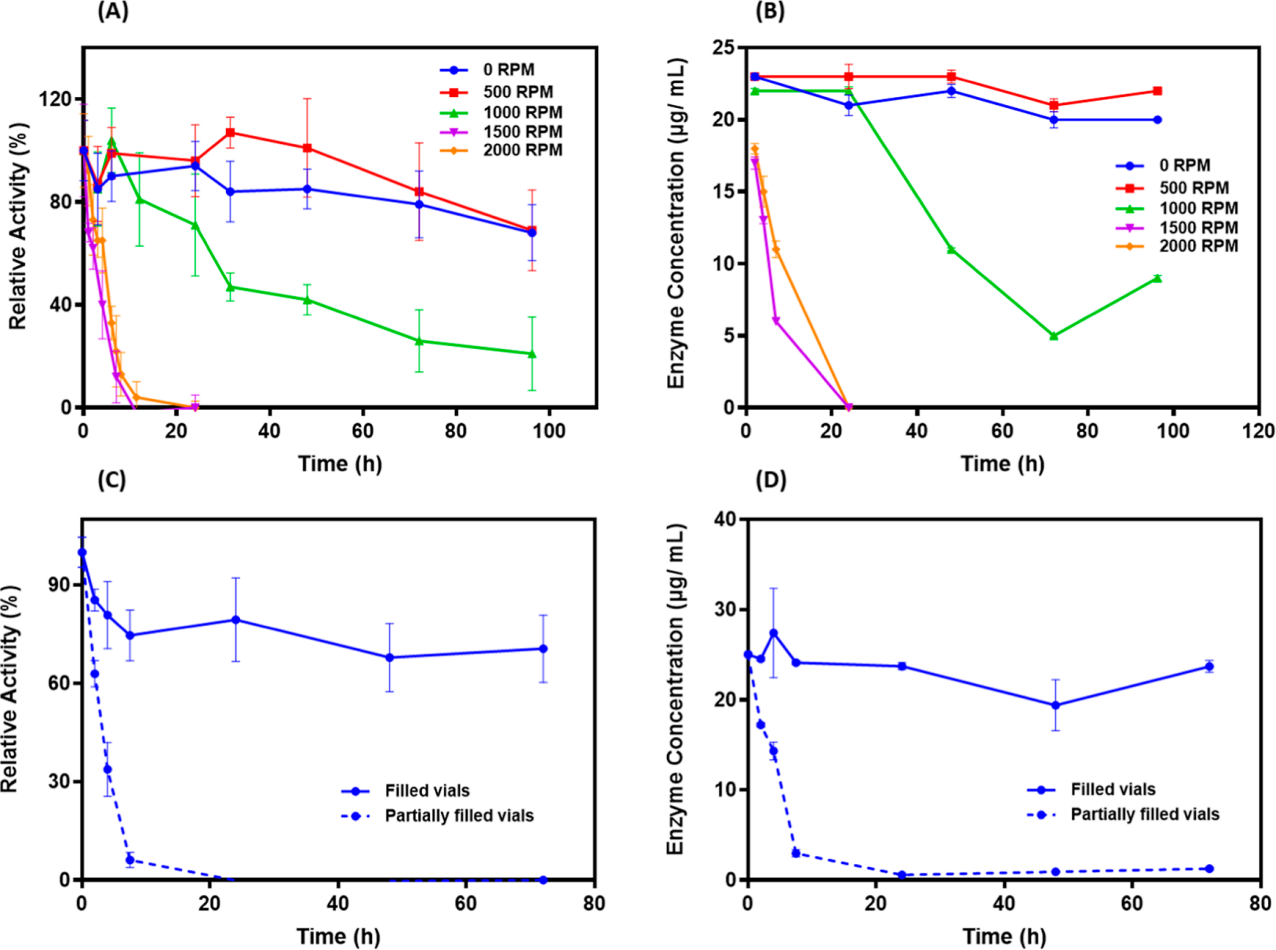
Impact of mixing rate on (A) relative enzyme activity and (B) enzyme concentration in the absence of PET. Impact of mixing for samples with and without head space on (C) relative enzyme activity and (D) enzyme concentration in the absence of PET. Experiments performed using 1000 nM Fast-PETase at 45 °C in polypropylene vials. Error bars indicate the standard deviation of experiments performed in triplicate.

In the absence of PET films, the loss of enzyme activity is most likely due to adsorption at the air–water interface or shear-induced unfolding as a result of mixing. To test if the presence of the air–water interface is a driver for protein inactivation, mixing experiments were performed using completely filled polypropylene tubes to minimize the presence of the air–water interface. Similar to the previous experiments, FAST-PETase (1 μM) was mixed at 1000 rpm and samples were removed periodically and assayed for residual enzyme activity and protein concentration. The partially filled tubes lost enzyme activity over 6–12 h, which also tracks with the loss of protein from solution (Figure 2C,D). Interestingly, under the conditions where the vials were filled to minimize the air–liquid interface, no loss of enzyme activity or soluble enzyme was observed over a period of almost 3 days. This supports the hypothesis that the presence of the air–water interface is the primary driver of activity loss resulting from protein aggregation and precipitation from solution (Scheme 1). To better understand how the tube material type contributed to PETase inactivation, experiments were conducted using partially and completely filled glass HPLC vials. For the partially filled HPLC vials with mixing, the rate of deactivation of FAST-PETase was considerably slower and corresponded with the loss of enzyme from solution (the half-life of FAST-PETase in glass and polypropylene vials was 18 and 3 h, respectively). Conversely, for completely filled HPLC, no significant loss of enzyme activity over 72 h while mixing at 1000 rpm was observed (Figure S5).

**Scheme 1:**
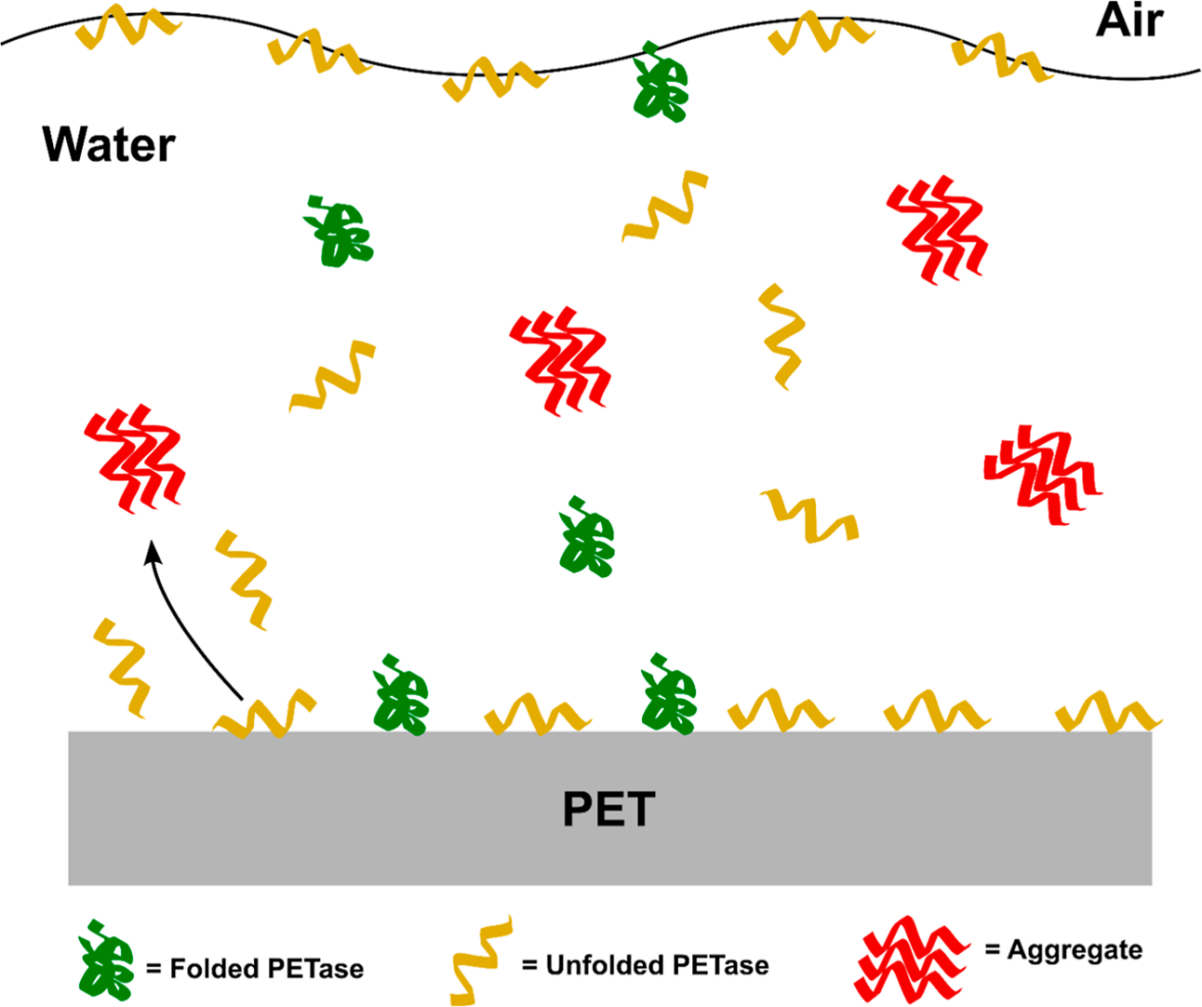
PETase Interaction with the Air-Liquid Interface and the PET-Liquid Interface Leads to Unfolding and Aggregation

Vigorous mixing, while often advantageous in bioreactors, can be detrimental to enzymes due to their sensitivity to shear and interfacial stress at both air–water and solid–liquid interfaces. Enzyme sensitivity to shear and mixing has previously been observed with cellulase,^64,65^ NADH oxidase,^66,67^ dextransucrase^68^ and lipase.^69^ Similarly, protein aggregation and loss from solution during mixing has been reported for insulin,^70,71^ antibodies,^72^ and bovine serum albumin (BSA).^54^ Notably, Schvartz and co-workers showed that proteins, including porcine hemoglobin, alpha-synuclein, and BSA, were generally more stable under nonmixed than mixed conditions when studied in containers comprised of glass, Teflon, and polypropylene.^54^ Studies with therapeutic proteins have shown that proteins adsorb at the air–water interface resulting in protein unfolding and gelation at the interface.^59,73–76^ Mixing, in particular, can cause the formation and collapse of bubbles, which form transient air–liquid interfaces to which proteins may absorb. Upon the collapse of such bubbles, denatured protein molecules may be released back into the bulk solution, which in turn can seed aggregation. An alternating production and reduction of interfacial area can also occur due to agitation or stirring in the presence of headspace.^59^ Similarly, bubbles that are formed during mixing may coalesce with the air–liquid interface at the surface of the bulk solution, which may also release denatured protein molecules. The introduction of shear stresses as a result of mixing may further drive aggregation by inducing denaturation even in the absence of air–liquid interfaces. As mixing speed increases, these effects are presumably more pronounced, which is consistent with our findings with PETase. Our observation that FAST-PETase inactivates more quickly in polypropylene tubes than in glass tubes may be due to partial unfolding of FAST-PETase by the more hydrophobic polypropylene surface. The partially unfolded enzyme, which may desorb from the tube wall back into solution, will be more likely to bind to the hydrophobic air–water interface where it may be further destabilized by mechanical forces from mixing (Scheme 1).^54^

### Increase in PET Surface Area Leads to More Rapid PETase Inactivation

Our findings clearly suggest that interactions with the hydrophobic air–liquid interface promotes PETase inactivation. Due to the hydrophobicity of PET, there is a possibility that PETase-PET surface interactions may also contribute to PETase unfolding and aggregation (Scheme 1). If so, we would expect that increasing the surface area of PET should promote PETase inactivation. To assess this hypothesis, we varied the exposed PET surface area by increasing the number of films added to the vials. In this case, we again minimized the presence of the air–water interface by filling the vials completely. The results of depolymerization studies indicated that the initial rate of PET degradation was directly proportional to the exposed PET surface area (Figure S6). This is expected since under these conditions, there is more enzyme than exposed binding sites so the rate of the enzyme–substrate complex formation is dependent on the surface area. Interestingly, after ∼24 h, the depolymerization rate of the reactions consisting of three or more PET films significantly declined, unlike at short times, and eventually plateaued at ∼30 h (Figures 3, and S6). Conversely, the reaction with one film continued to depolymerize PET up to 55 h with only a slight decrease in rate. The continued degradation of PET beyond 48 h in the sample with one film was likely due to the enhanced stability of the enzyme in the absence of the air–water interface (Figure 2). These results clearly demonstrate that the rate of decrease in the enzyme catalyzed PET degradation reactions is proportional to the PET surface area in the reaction.

**Figure 3:**
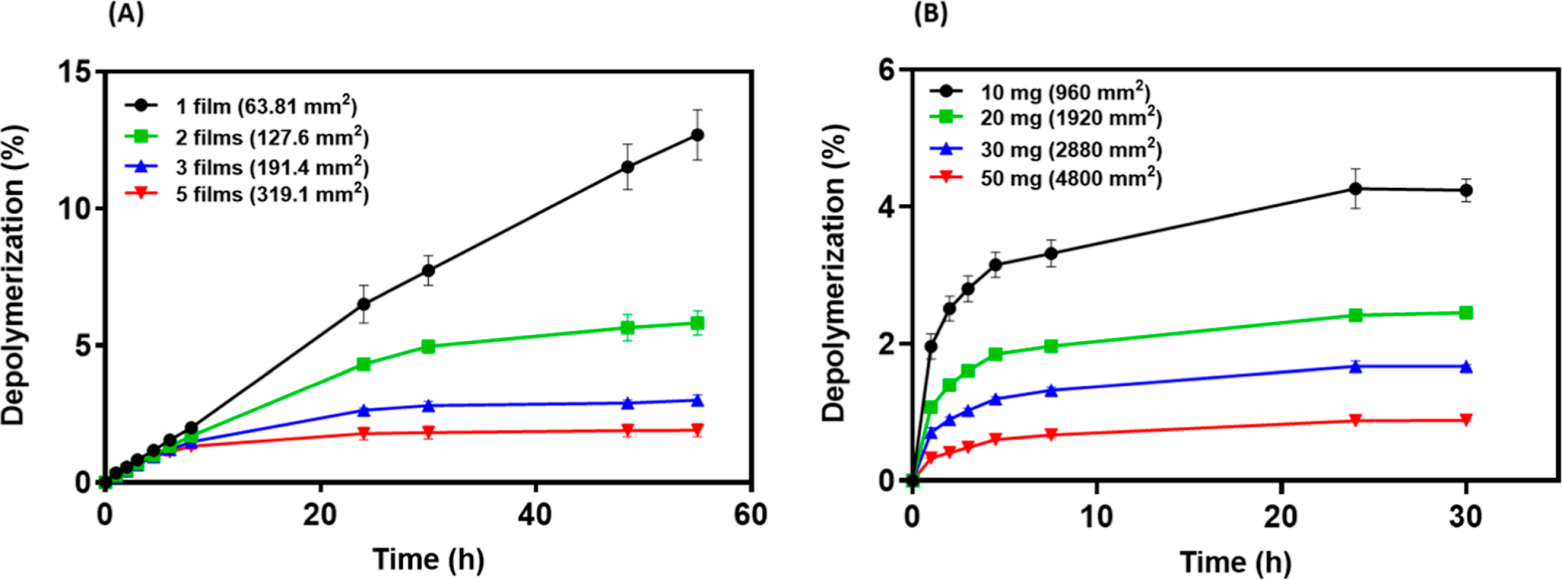
Impact of varying PET surface area on PET degradation (200 nM Fast PETase, 1000 rpm, pH 8, 45 °C, filled tubes), (A) varying the number of amorphous (*X*_c_ = 2%) PET films (1 film (∼10 mg) equates to specific surface area of 6381.36 mm^2^/g), (B) varying the amount of PET microparticles (*X*_c_ = 37.7%) (∼10 mg equates to specific surface area of 0.1 m^2^/g). Error bars indicate the standard deviation of experiments performed in triplicate.

Figure 3B shows a similar experiment where the PET exposed surface area was varied by adding different amounts of microcrystalline PET microparticles. Under these conditions, the exposed PET surface area is three- to twenty-times higher than that used in the experiments with five PET films. Over the first few hours, PETase showed good activity on the microcrystalline particles; however, by 5 h, the rate of depolymerization began to decline rapidly and practically no more conversion was observed by ∼8 h for all samples independent of the mass of microparticles. It is important to note that the crystallinity of the microparticles was considerably greater than the films (37.7% for the microparticles vs 2% for the films). Notably, even under nonmixed conditions, the rate of PET degradation declined for conditions with high surface area for both amorphous films and PET microparticles (Figure S6). Under the nonmixed conditions, the air–liquid interface and shear stress are significantly minimized and the main contributor to enzyme inactivation is the PET surface area. This provides further evidence that the PET surface area is a potentially significant contributor to PETase inactivation during PET depolymerization. A similar phenomenon was observed by Sluzky et al. where the rate of insulin aggregation increased with increasing Teflon surface area during shaking experiments.^70,71^

Understanding the impact of increasing surface area of PET on enzymatic degradation is a critical factor for successful scale-up of enzymatic recycling due to the need to balance the high energy costs associated with reducing the PET particle size and the possible reaction rate enhancement typically expected with decreased particle size. As pointed out by Wei et al., an increase in PET degradation is not always observed with an increase in surface-to-volume ratio of the PET.^77^ As they note, previous reports have suggested that such reductions in the extent of depolymerization with small particles may be due to strain-induced crystallization during the micronization processes.^77–79^ While plausible, it is also likely that such reductions are the result of an increase in the unfolding of PETase as a result of the increase in surface area of PET. Currently, it is unclear if there is a difference in how PETase interacts with amorphous and crystalline surfaces and if either of these surfaces is more detrimental for PETase stability. Another possible explanation for this observation is the adsorption of the enzyme onto the PET surface and formation of an irreversible enzyme–substrate complex which results in enzyme inactivation. However, given the low total surface area of PET in our film experiments, this effect is likely limited but could be significant for the samples containing higher concentrations of microplastics due to the large interfacial surface area.

### Surface Active Additives Prevent Adsorption and Inactivation of PETase at Interfaces

When working with the low nanomolar solutions of PETases as is often reported for PET degradation reactions, great care is required to prevent the loss of activity.^25,28,33,34^ For example, if enzyme solutions were prepared by serial dilution, the rate of soluble activity degradation was not proportional to enzyme concentration (Figure S7A,B). This problem is commonly observed when working with surface active proteins at low concentrations^80^ due to the protein sticking to surfaces such as pipet tips and tube walls. Similar issues with nonspecific adsorption of cutinases were reported by Badino and co-workers when preparing protein standards for adsorption measurements, which led them to suggest using low-binding tubes and plates for these studies.^41^ A classic approach to prevent loss of enzyme by nonspecific adsorption to surfaces is to add a blocking agent to passivate the surface and block strong adsorption sites.^80^ Gelatin, BSA and water-soluble polymers such as PEG 8000 have been demonstrated to be effective as blocking agents.^80^ In order to determine if PEG 8000 was an effective blocking agent to prevent PETase from adsorbing to surfaces, we prepared buffers with 0.1% w/v of PEG 8000 and prepared serial dilutions of the PETases. Indeed, the addition of PEG 8000 helped to prevent enzyme loss from solution and resulted in linear reaction curves for FAST and HotPETase concentration when measured with pNA (Figure S7A,B).

Since the addition of PEG 8000 was effective in preventing loss of PETase by nonspecific adsorption to glass and plastic surfaces during pipetting, this suggests that PEG 8000 and other blocking agents may also be effective in preventing the enzyme from adsorbing to and unfolding at the hydrophobic air–water and PET-water interfaces. A similar problem with protein unfolding at interfaces occurs with therapeutic proteins where aggregation and formation of potentially immunogenic particles occur due to agitation and contact with surfaces during mixing, pumping, shipping and handling of the proteins.^48,49,54,59,81^ It is common to add nonionic surfactants to liquid protein formulations to protect the proteins from aggregation at air–liquid interfaces.^72,82^ Sluzky et al. found that insulin aggregation at hydrophobic interfaces could be prevented by addition of nonionic surfactants such as Tween 20 and Brij 35 at concentrations as low as 0.5 the Critical Micelle Concentration (CMC). They also found polymeric surfactants such as Pluronic F68 and F127 were effective at two times the insulin concentrations.^70^ In studies with *Trichoderma reesei* cellulase, the addition of surface-active additives such as BSA and Tween 20 were found to reduce inactivation and precipitation of low concentrations of the enzyme at the air–water interface during mixing leading to increased cellulose conversions.^64,65^ Similarly, the addition of surfactants have been demonstrated to protect endoglucanase from inactivation at the air–water interface and by nonspecific adsorption to lignin.^83^ Based on these findings, we performed a mixing study to screen a variety of nonionic surfactants, polymers, solvents and proteins that have been previously found to be effective in protecting proteins from inactivation at surfaces.

Enzyme mixing stability studies were performed at conditions similar to our previous experiments (200 nM FAST-PETase, 45 °C, partially filled tubes) but were mixed at high mixing rates (2000 rpm) without PET. Enzyme samples were removed periodically for assay using pNA and the activity was compared to the initial activity of PETase added. Under these conditions, FAST-PETase was completely inactivated within 4 h if no stabilizer was added to the buffer. Remarkably, we identified a variety of compounds that were effective in preventing inactivation of the enzyme for over 92 h under these high mixing rates (Figure 4). Of the small nonionic surfactants screened, Triton X-100 and Brij 35 worked well to protect the enzyme at half of their respective CMCs. The polymeric surfactant Pluronic F127, along with 0.5% w/v PEG 8000, 0.5% w/v gelatin and 1 mg/L BSA were also effective in preventing activity loss. Interestingly, a protective effect was also observed upon addition of 10% (v/v) DMSO, which may be due to the solvent-induced alteration of the surface tension of the PET-liquid interface. Notably, the inclusion of DMSO has been reported previously to improve PET degradation.^28,34^ Although not studied rigorously, previous reports of enhanced stability upon addition of the cell lysing agent BugBuster, which is composed of a mixture of nonionic and zwitterionic surfactants, and given our results is also likely protective toward inactivation.^26^ A similar stabilization effect may explain the positive enhancements observed by the addition of PEG to reactions containing cutinase for hydrolysis of PET.^46^ While DMSO prevented rapid inactivation of FAST-PETase due to mixing, the enzyme activity appears to decrease with time, likely due to unfolding as a result of solvent interactions between the DMSO and hydrophobic amino acid side chains.^84,85^ In contrast, PETase incubated with Triton X-100, Brij 35, and Pluronic F127, was significantly more stable. This may be due to the ability of amphiphilic surfactants to inhibit aggregation of unfolded proteins and assist in protein refolding.^86–88^

**Figure 4:**
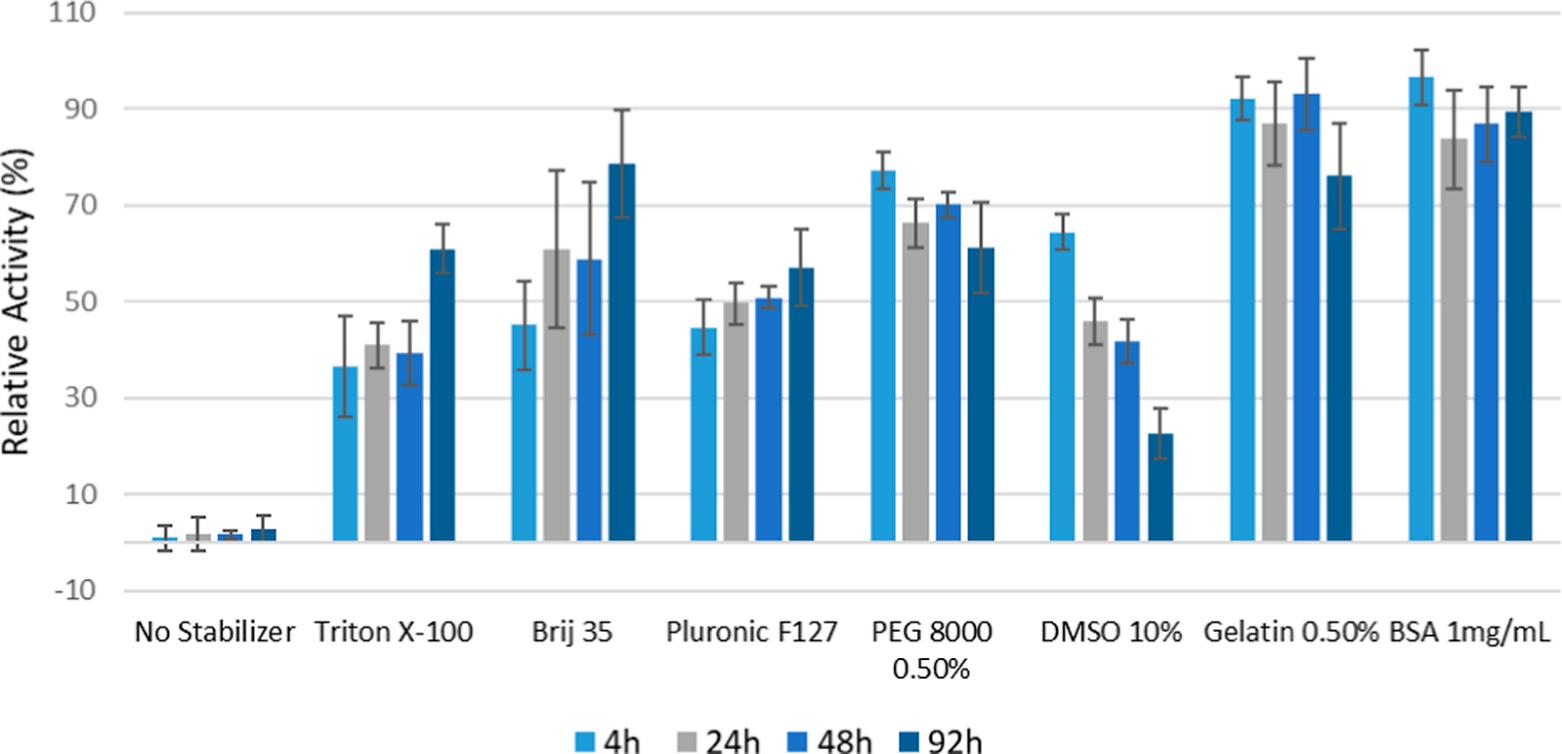
Various stabilizers reduce FAST-PETase inactivation during mixing (200 nM FAST- PETase, 2000 rpm mixed, 45 °C, no PET). Error bars indicate the standard deviation of experiments performed in triplicate.

In addition to conventional stabilizers, we also examined the agitation-induced deactivation of FAST-PETase when tethered to mixed lipid bilayers in the form of small unilamellar vesicles (SUVs), which have been shown to suppress and even reverse protein misfolding and aggregation.^89–94^ Negatively charged bilayers were prepared by combining the anionic lipid 1,2-dioleoyl-*sn*-glycero-3-phospho-(1′-rac-glycerol) (DOPG) with the zwitterionic lipid 1,2-dioleoyl-*sn*-glycero-3-phosphocholine (DOPC), whereas positively charged bilayers were generated by mixing the cationic lipid 1,2-dioleoyl-*sn*-glycero-3-ethylphosphocholine (DOEPC) with DOPC. His-tagged FAST-PETase was tethered to the SUV surface via Ni–histidine interactions with DGS-NTA­(Ni) lipids incorporated into each bilayer at 5% (w/w). Across all lipid bilayer compositions, tethered FAST-PETase retained significantly more activity during mixing than in its soluble form (Figure S8). Consistent with prior studies showing that stabilization is greatest when the bilayer and enzyme share the same sign of charge, positively charged SUVs were most effective, presumably by both blocking the air–water interface and allowing transient, noncovalent interactions between enzyme and bilayer without excessive attraction that could cause surface denaturation.^92–94^

To determine if additives that maintain PETase stability during mixing could also enhance PET degradation, we tested two stabilizers identified in the preliminary screening assays, Brij 35 and PEG 8000. For this, we used Brij 35 as a representative small molecule additive and PEG 8000 as a representative large molecule stabilizer; both were selected since they are potentially practical for use at large scale. While gelatin and BSA both showed better stabilization in our studies, they were not tested since recent work suggests that excess added protein can inhibit PET degradation.^95^ We performed degradation studies using FAST-PETase with various concentrations of these stabilizers under a high mixing rate (2000 rpm). Although both additives successfully protected the enzyme from inactivation, they also significantly hindered the overall process of PET degradation (Figures S9 and S10). For Brij, at 1:100 of the CMC we observe the expected mixed result of some stabilization due to low surfactant concentration but also enhanced degradation due to some interaction with PET. Unfortunately, none of the tested stabilizer concentrations improved PET degradation beyond what was observed in simple, nonmixed samples, indicating that while these agents preserved PETase activity, they also interfered with the enzyme’s ability to degrade the plastic.

These data suggest that while the Brij surfactant and PEG 8000 effectively prevent the enzyme from binding to the air–water interface and being inactivated, it is likely that the presence of the additives also blocked the PET interface, preventing the enzyme from binding productively to the PET to enable catalysis (Scheme 2). This is likely the same phenomenon recently observed by Alper’s group looking at the impact of extraneous protein fouling of PET surfaces and preventing degradation by PETase.^95^ In their study, they looked at the ability of different laboratory and household contaminants to inhibit PET degradation. They found that proteins, such as BSA and those found in cell culture media, were able to bind to PET surfaces and slow PET degradation. The effect was worse if the protein contaminants were denatured. They speculated that the inhibition of PET degradation is likely due to a decrease in the PET surface accessibility; however, they note that there may be some more complex interactions leading to PETase denaturation when in contact with the fouling agents. In light of our results, it seems most likely that the fouling proteins are adsorbing to the PET interfaces and blocking surface accessibility to the PETase; an analogous effect is likely for the other stabilizers such as surfactants and polymers. A similar effect was reported with endoglucanase where addition of Tween 20 was observed to reduce the adsorption of the enzyme to cellulose.^96^ This suggests an interesting balance between stabilizing the enzyme to prevent unfolding at surfaces while promoting binding of the folded protein at PET surfaces for effective surface reaction. Furthermore, this suggests that if one could identify a passivating agent that selectively binds to the air–liquid interface, but not the PET-liquid interface, it may be possible to dramatically improve PETase-catalyzed PET hydrolysis.

**Scheme 2:**
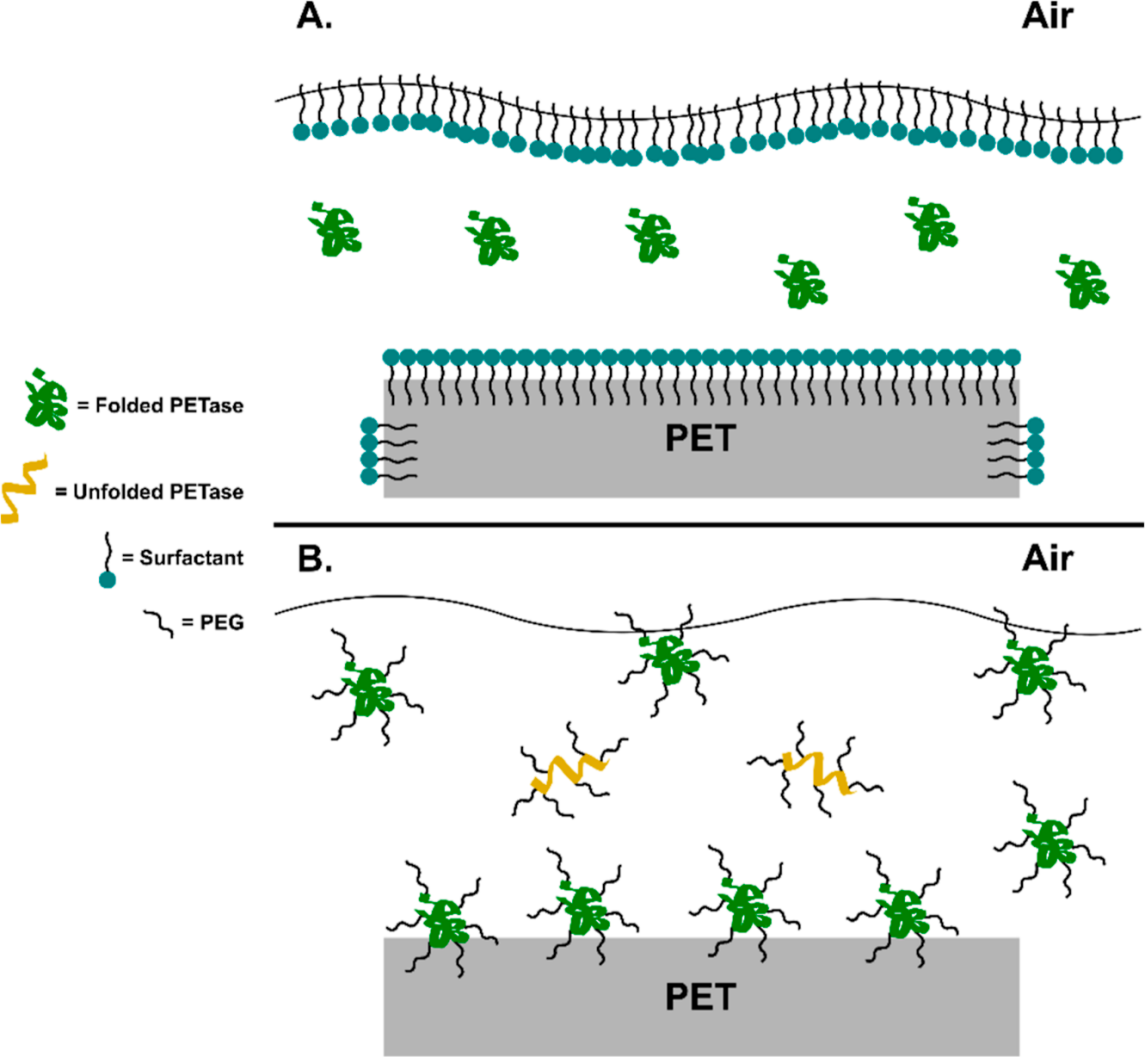
(A) PETase Unfolding is Minimized at the Air-Liquid Interface by the Presence of Surfactants, However the Surfactant Also Blocks the PET Surface Preventing Productive PETase Binding to the PET; (B) PEGylation of PETase Prevents Aggregation of Unfolded PETase while Allowing Productive Binding of PETase to the PET Surface

### PEGylation of PETase Prevents Inactivation during Mixing

Another approach that has been demonstrated to modulate protein adsorption and aggregation at interfaces involves PEGylation.^97–100^ PEGylation is widely used in the context of therapeutic proteins to enhance colloidal and interfacial stability by providing a steric barrier to aggregation. Steric repulsion provided by the PEG chains attached to the protein’s surface are thought to reduce interfacial gelation and prevent aggregation.^99^ Moreover, in some cases, PEGylation can alter the hydrophilic–lipophilic balance of proteins, thereby reducing the surface affinity of the construct. Studies looking at improving PETase activity and stability by PEGylation^101^ and modification with polymers produced by atom transfer radical polymerization^102^ have demonstrated modest increase in activity or stability. However, the protection of enzymes by polymer modification to mixing-induced inactivation has yet to be explored.

Given the precedent of stabilizing proteins at interfaces via PEGylation, we investigated whether the modification of PETase with PEG may prevent PETase inactivation to mixing. For this, we modified FAST-PETase with a 5000 Da PEG using a commercially available PEG-SPA at a molar ratio of 25 PEG-SPA to 1 enzyme primary amine. Successful modification of the enzyme was verified by SDS-PAGE (Figure S11) where the complete disappearance of the native enzyme band indicated nearly 100% modification of the available enzyme with PEG. PEG modified enzymes appeared as a smear at higher molecular weights. Since the presence of free PEG could negatively impact PETase adsorption to PET, as we observed with the free PEG 8000, PETase was purified using nickel affinity chromatography to remove the unreacted PEG. Removal of the unreacted PEG was verified by iodine staining of the SDS-PAGE gel (Figure S11). Although the modification of PETase was nonspecific (i.e., via reaction of random primary amines), FAST-PETase retained 75% ± 5% of its initial (i.e., unmodified) activity on pNA.

While the PEGylated PETase showed good activity with the small pNA substrate, polymer modification may inhibit activity against larger substrates due to steric blocking of the active site.^103^ This is especially of concern when using nonspecific attachment chemistries such as PEG-SPA, which can potentially react with any free primary amine on the enzyme surface. Analysis with PRELYM^104^ predicted that four of FAST-PETase’s nine available primary amines were likely to react. The *N*-terminus and lysine residue 233 were identified as being fast-reacting sites (Table S2) where the *N*-terminus is located on the far side of the enzyme away from the active site and residue 233 is located on the side of the enzyme closer to the active site (Figure S12). To determine if the PEGylated PETase was active against PET and stable while mixing, solutions were prepared with PEGylated PETase at a concentration with the same volumetric enzyme activity that was equivalent to 200 nM of unmodified PETase (based on activity with pNA) and degradation of amorphous PET films was monitored at 45 °C and 2000 rpm mixing using the PEGylated and non-PEGylated enzyme. Under these conditions, the unmodified FAST-PETase was completely inactivated within a few hours and produced less than 0.5 mM of soluble TPA equivalents. Impressively, the PEGylated enzyme continued to degrade PET while mixing at 2000 rpm where more than 50% of the film was degraded by 125 h (Figure 5) when the reaction was terminated due to an observed decrease in solution pH. These results clearly demonstrate that polymer modification of PETase is an effective approach to prevent surface inactivation of the enzyme while also maintaining activity against the solid substrate (Scheme 2). Given that all the PETase was modified with PEG (i.e., no residual unmodified enzyme was present), the observed activity can be attributed to PETase with at least one PEG attached. Furthermore, the observation that the PEGylated enzyme degrades PET indicates that the active site is accessible and the PEG chains are not preventing PET binding and turnover.

**Figure 5:**
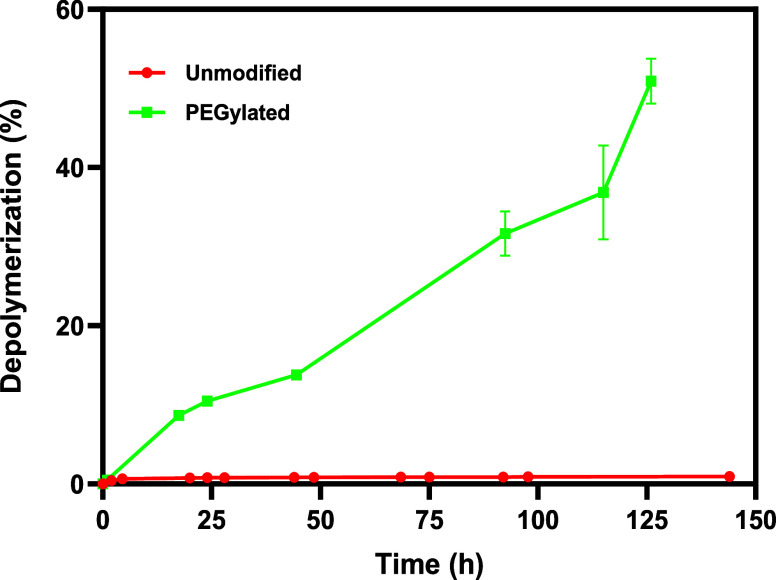
Degradation of amorphous PET by unmodified and PEGylated FAST-PETase while mixing at 2000 rpm and 45 °C. Error bars indicate the standard deviation of experiments performed in triplicate.

While PEGylation likely reduces interactions of PETase with the PET surface, this effect is compensated by improved interfacial stability, leading to higher overall conversion of PET over longer times. We hypothesize that the enhanced PET hydrolysis observed at high mixing rate (2000 rpm in this case) results from the PEGylated enzyme’s resistance to surface induced unfolding and aggregation. Such resistance to unfolding and aggregation may be due to the shielding of hydrophobic residues on the surface of PETase by PEG. By shielding hydrophobic residues, the enzyme will be less prone to absorb and denature on the surface of the vessel and the air–water interface. Additionally, shielding of hydrophobic patches on the surface of the enzyme may prevent hydrophobic protein–protein interactions that drive aggregation.

## Conclusions

In this work, we investigated *Is*PETase and its variants under mixed and nonmixed conditions while monitoring depolymerization efficiency and enzyme activity. Consistent with previous reports, all PETase variants were inactivated within 12 h under high mixing in the presence of the air–liquid interface. Inactivation of PETase tracked with loss of soluble enzyme from solution as well as the formation of insoluble aggregates. No clear correlation between sensitivity to interfacial inactivation and the *T*_m_ of the enzyme were observed for FAST and HotPETase. More detailed studies are needed to determine the relative sensitivities of each enzyme to the air–water and solid–water interface and their relationship to intrinsic properties of the enzyme such as the *T*_m_. Nevertheless, by reducing the air–liquid interface, we demonstrated that the stability of the enzyme may be significantly enhanced. Likewise, by preventing adsorption of PETase on the surface of PET films, enzyme activity was retained over longer times. Although blocking agents that prevented surface adsorption improved stability, they adversely impacted activity on PET. In contrast, PEGylation was effective at maintaining enzyme activity and improving degradation efficiency. We are currently planning comprehensive studies to understand the effects of PEGylation degree and location on PETase activity and deactivation kinetics. Furthermore, similar studies with cutinases and other PET degrading esterases may provide further insight into the nature of the inactivation of these enzymes by surface interactions and may enable the development of rational strategies to improve the performance of PETase and similar enzymes for degradation of plastics under industrially relevant conditions.

## Supplementary Material



## Notes

Information including results from additional PET degradation studies for FAST-PETase, HotPETase and *Is*PETase, *T*_m_ determination of PETase variants, Coomassie staining of PETase aggregates, FAST-PETase activity retention when tethered to SUVs, SDS-PAGE of PEGylated FAST-PETase and PRELYM analysis of PDB 7SH6 are found in the Supporting Information (PDF)
