# Supplementary material for: Inactivation of PETase at Interfaces Inhibits PET Plastic Depolymerization
Source: ACS Sustain Chem Eng. 2026 May 20;14(21):9741–54. doi: 10.1021/acssuschemeng.5c13215 (PMC13231421; doi:10.1021/acssuschemeng.5c13215)
Supplement: Supplementary file 1 [file sc5c13215_si_001.pdf]

Supporting Information

Inactivation of PETase at Interfaces Inhibits PET Plastic Depolymerization

Alecia Robinson[2, †], Hannah Lippincott[2, †], Chris E. MacFarlane[1], David J. Kelaita[3], Catherine Moul[1], Gur Pines[3,4], Daniel K. Schwartz[3], Joel L. Kaar[3], Jason T. Boock[1], Jason A. Berberich,[1,2,]*

[1] Department of Chemical, Paper and Biomedical Engineering, Miami University, Oxford, OH, 45056, USA

[2] Department of Chemistry and Biochemistry, Miami University, Oxford, OH, 45056, USA

[3] Department of Chemical and Biological Engineering, University of Colorado, Boulder, CO, 80309, USA

[4] Institute of Plant Protection, Agricultural Research Organization − Volcani Institute, Rishon LeZion, 7505101, Israel

[†] These authors contributed equally to this work

***Corresponding Author:**
Dr. Jason Berberich
650 E. High St.
Engineering Building 64
Oxford, OH 45056
berberj@miamioh.edu

Number of pages: 14

Number of tables: 2

Number of figures: 12

Supporting Information Table of Contents:

Supporting Tables

**Table**	**Contents**	**Page**
Table S1.	Summary of average TPA_{eq} and percent depolymerization of PET by 200 nM Fast PETase under the various reaction conditions tested.	S3
Table S2.	Results from PRELYM analysis of FAST-PETase (PDB: 7SH6)	S4

Supporting Figures

**Figure**	**Contents**	**Page**
Figure S1.	Degradation of PET and activity of FAST-PETase, HotPETase and isPETase	S5
Figure S2.	Thermal denaturation of PETase variants.	S7
Figure S3.	Coomassie stain of isPETase aggregated on vial walls after mixing	S8
Figure S4.	Impact of mixing rate on the activity of 200 nM FAST-PETase	S9
Figure S5.	Impact of mixing on samples with and without head space in glass vials	S10
Figure S6.	Impact of varying surface area of amorphous PET and PET microparticles on PET degradation efficiency	S11
Figure S7.	Impact of PEG on linearity of pNA hydrolysis rate vs enzyme concentration	S12
Figure S8.	FAST-PETase activity retention when tethered to the respective SUVs	S13
Figure S9.	PET degradation and relative enzyme activity in the presence of PEG 8000	S14
Figure S10.	PET degradation and relative enzyme activity in the presence of Brij 35	S15
Figure S11.	SDS-PAGE and iodine stain gel of FAST-PETase after PEGylation	S16
Figure S12.	Structure of FAST-PETase (PDB ID 7SH6).	S17

Table S1: Summary of average TPA_{eq} and percent depolymerization of PET by 200 nM Fast PETase under the various reaction conditions tested.

Data corresponding to figure	Temperature (°C)	Mixing speed (rpm)	Vial fill	Substrate	mM TPA_{eq} at 24 hrs	mM TPA_{eq} at 48 hrs	Depolymerization (%) at 24 hrs	Depolymerization (%) at 48 hrs	Comments
Figure 1A	40	0	⅔ fill	1 PET film	2.61	4.44	5.38	9.15	X_c = 2%
	40	1000	⅔ fill	1 PET film	1.20	1.17	2.48	2.41	
	45	0	⅔ fill	1 PET film	3.36	5.52	6.93	11.4	
	45	1000	⅔ fill	1 PET film	1.37	1.45	2.83	2.98	
	50	0	⅔ fill	1 PET film	1.93	2.23	3.98	4.59	
	50	1000	⅔ fill	1 PET film	2.54	2.38	5.24	4.91	
Figure 3A	45	1000	Filled	1 PET film	3.10	5.52	6.51	11.5	
	45	1000	Filled	2 PET films	4.16	5.45	4.32	5.65	
	45	1000	Filled	3 PET films	3.83	4.21	2.64	2.90	
	45	1000	Filled	5 PET films	4.37	4.63	1.78	1.90	
Figure 3B	45	1000	Filled	10 mg PET micro-particles	2.01	N/A	4.26	N/A	X_c = 37.7%
	45	1000	Filled	20 mg PET micro-particles	2.31	N/A	2.41	N/A	
	45	1000	Filled	30 mg PET micro-particles	2.42	N/A	1.67	N/A	
	45	1000	Filled	50 mg PET micro-particles	2.16	N/A	0.87	N/A	
Figure 5	45	2000	⅔ fill	1 PET film	5.08	6.69	10.5	13.8	1:25 PETase-PEG
	45	2000	⅔ fill	1 PET film	0.384	0.404	0.791	0.834	Un-modified PETase
Figure S1A	45	1000	⅔ fill	1 PET film	2.19	2.31	4.52	4.75	X_c = 2%
Figure S8 A	45	2000	⅔ fill	1 PET film	1.00 for 0.05% free PEG	1.46 for 0.05% free PEG	2.06 for 0.05% free PEG	3.01 for 0.05% free PEG	0.005-0.5 % free PEG; X_c = 2%
Figure S9 A	45	2000	⅔ fill	1 PET film	1.03 for 1/20 CMC	1.78 for 1/20 CMC	2.13 for 1/20 CMC	3.67 for 1/20 CMC	1/100-1 CMC Brij; X_c = 2%

Note: Reactions were done in triplicate and values represent the average.

Table S1. Results from PRELYM analysis of FAST-PETase (PDB: 7SH6)

	Chain	Residue	Amino Group	ESA	pKa	Secondary Structure	H-Donor	Area Of Lower Charge	Interaction
**0**	A	29	N	1249.17	7.90		No		fast-reacting
**1**	A	95	LYS	0	10.32	Helix	No	Yes	non-reacting
**2**	A	148	LYS	0	9.94	Coil	Yes	Yes	non-reacting
**3**	A	177	LYS	0	8.99	Coil	Yes	Yes	non-reacting
**4**	A	227	LYS	143.92	9.35	Strand	Yes	No	slow-reacting
**5**	A	233	LYS	135.26	10.60	Coil	Yes	Yes	fast-reacting
**6**	A	252	LYS	0	11.04	Helix	Yes	Yes	non-reacting
**7**	A	253	LYS	33.33	7.22	Helix	Yes	Yes	non-reacting
**8**	A	259	LYS	139.03	8.49	Helix	Yes	Yes	slow-reacting

(A)

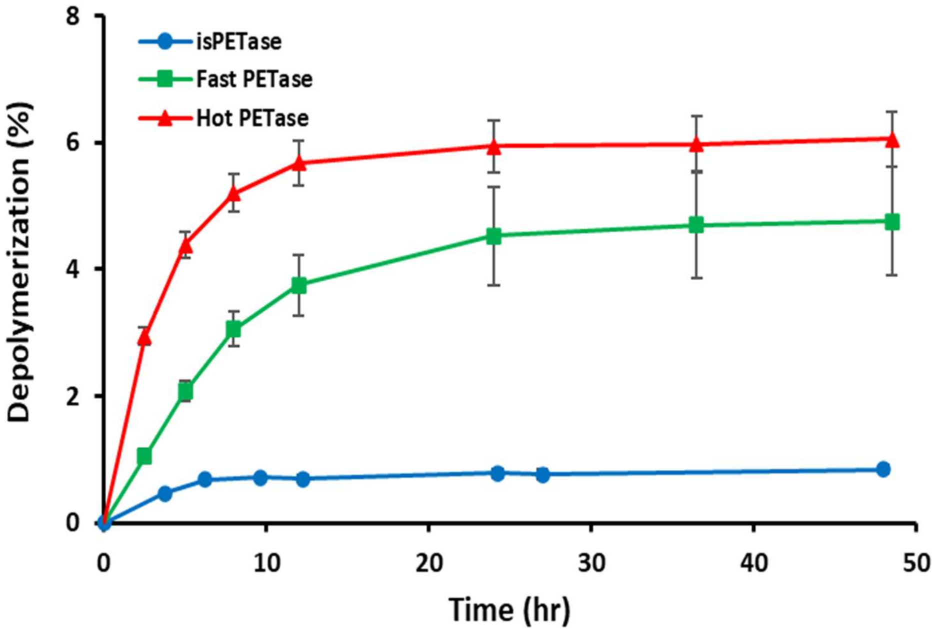

isPETase
Fast PETase
Hot PETase
Depolymerization (%)
8
6
4
2
0
0
10
20
30
40
50
Time (hr)

(B)

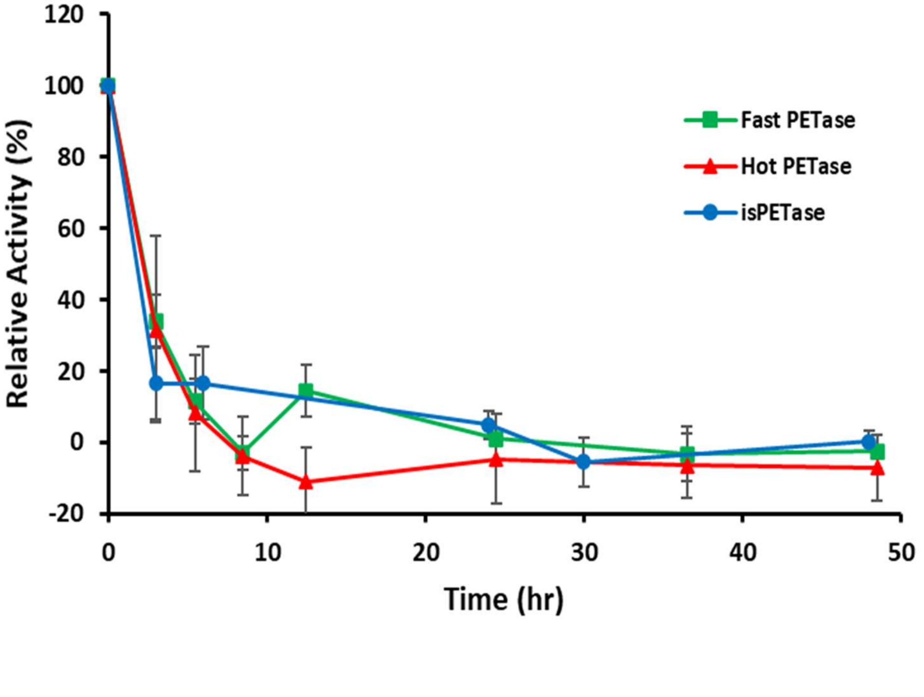

Fast PETase
Hot PETase
isPETase
Relative Activity (%)
120
100
80
60
40
20
0
-20
0
10
20
30
40
50
Time (hr)

(C)

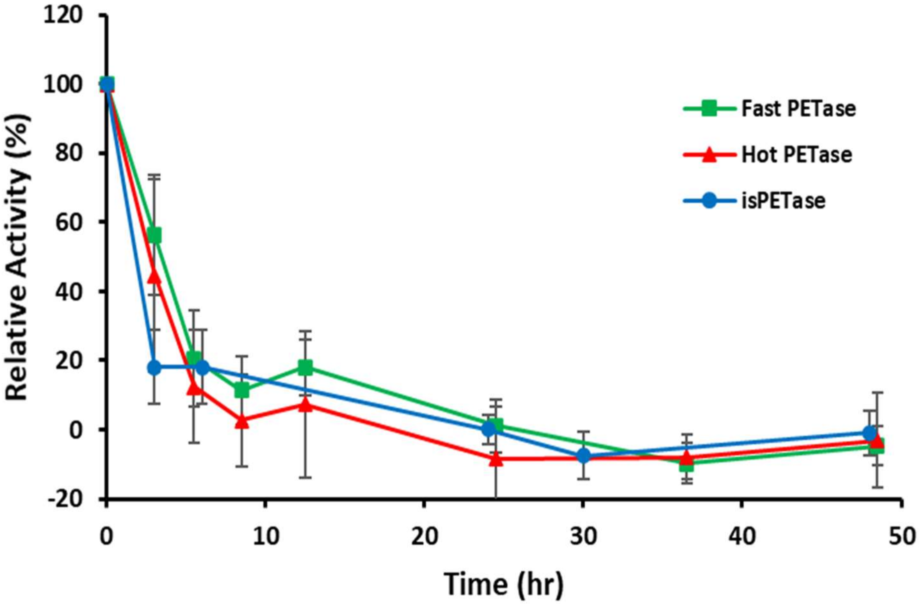

Figure S1. (A) Comparison of degradation of PET films by three variants of PETase. (B) Relative activity (%) of soluble PETase during reaction with PET films. (C) Relative activity (%) of soluble PETase with **no** PET films present. Error bars indicate the standard deviation of experiments performed in triplicate.

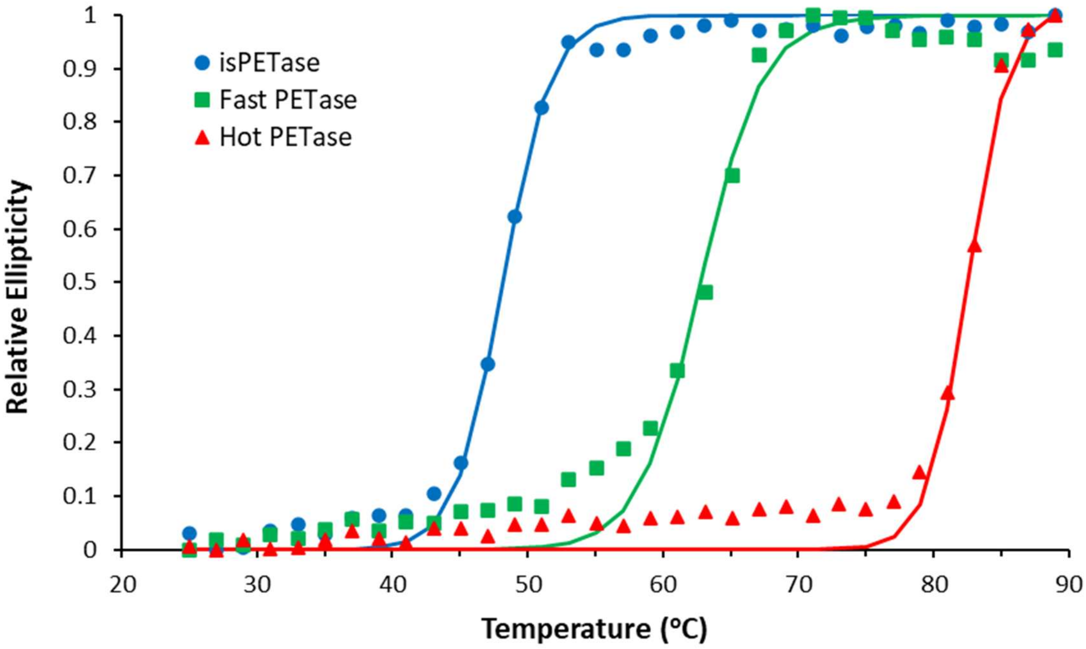

Figure S2: Thermal denaturation of PETase variants. Temperature was slowly ramped from 25ºC to 89ºC and ellipticity in millidegrees was measured at 220 nm for each temperature step. Mean residue ellipticity was calculated and is shown in circles. A thermodynamic model was used to fit the data to find the melting temperature (T_m). The model fit is shown as a solid line. The estimated T_m was found to be 48.2°C for isPETase, 62.8°C for FAST-PETase and 82.6°C for HotPETase.

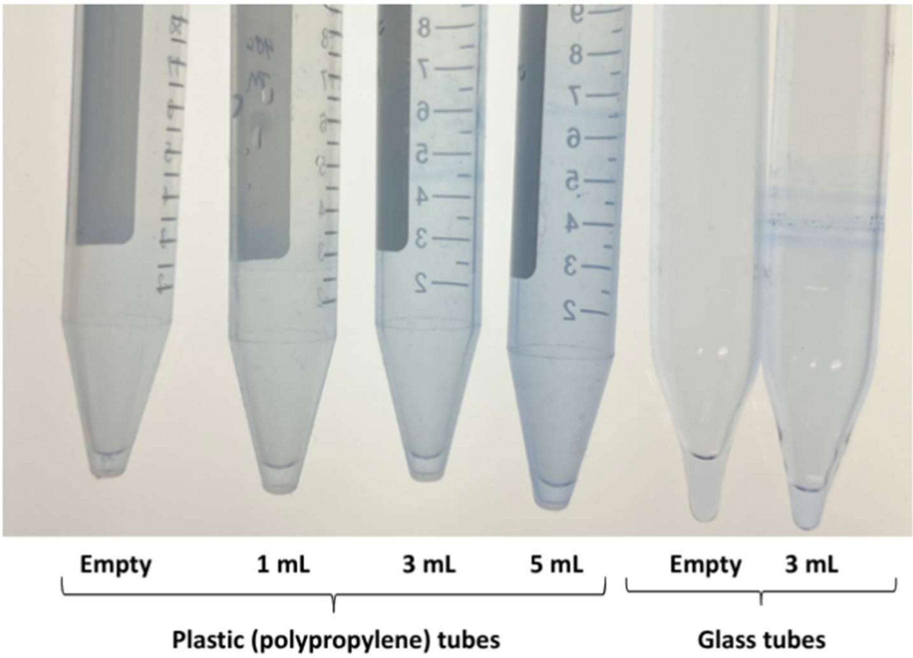

Figure S3. Coomassie stain of IsPETase aggregated on the walls of plastic and glass centrifuge vials after mixing. A different volume (0, 1, 3 or 5 mL) of 30µg/ mL IsPETase was added to each vial before mixing.

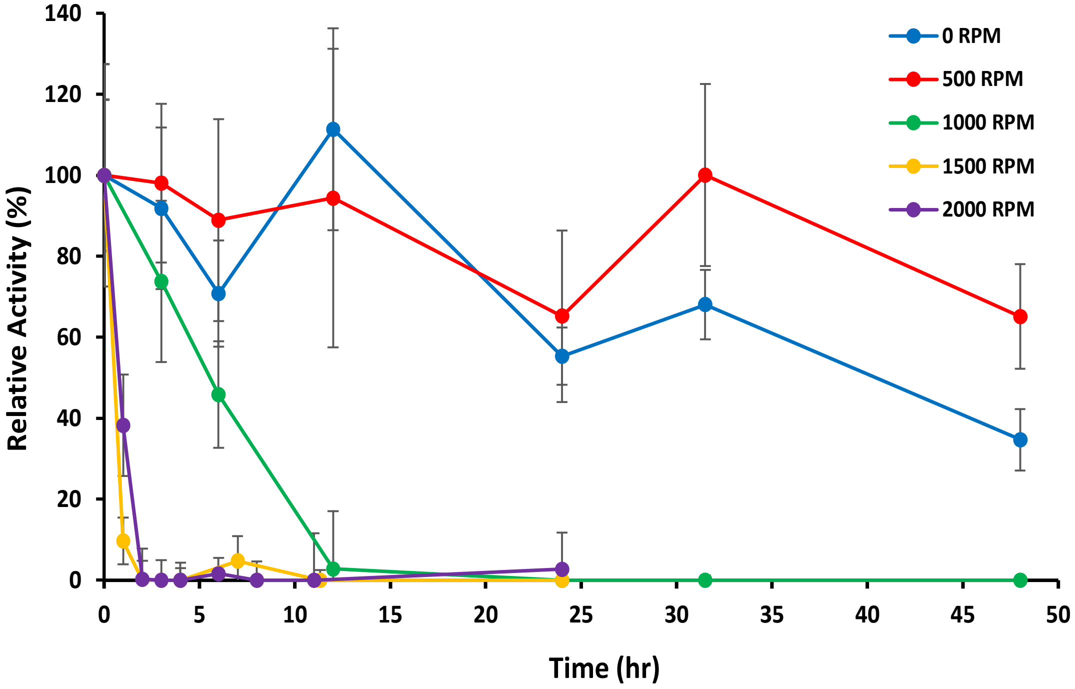

Figure S4. Impact of mixing rate on FAST-PETase activity (200 nM FAST-PETase, 45°C, 100 mM phosphate, pH 8.0). Error bars indicate the standard deviation of experiments performed in triplicate.

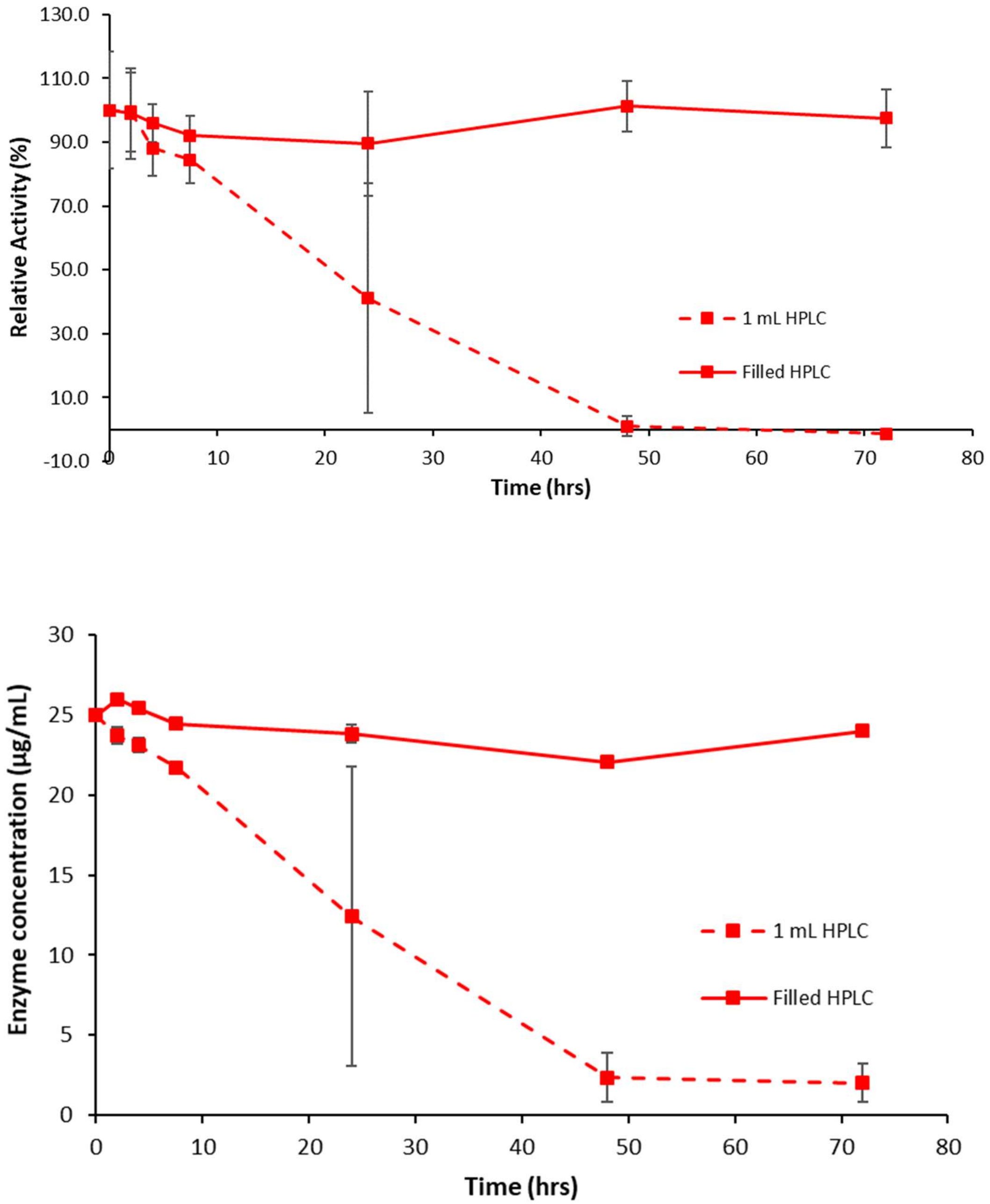

Figure S5: Impact of mixing for samples with and without head space on (A) relative enzyme activity and (B) enzyme concentration in glass vials. Experiments performed using 1000 nM Fast-PETase at 45°C. Error bars indicate the standard deviation of experiments performed in triplicate.

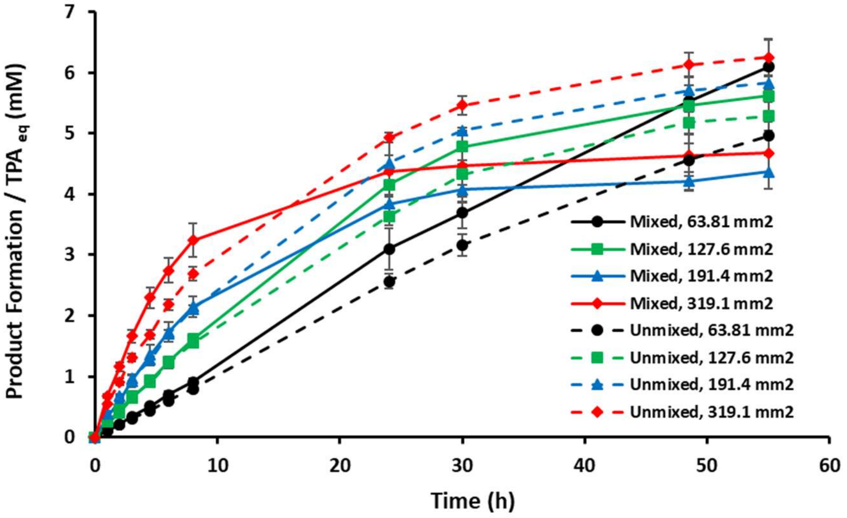

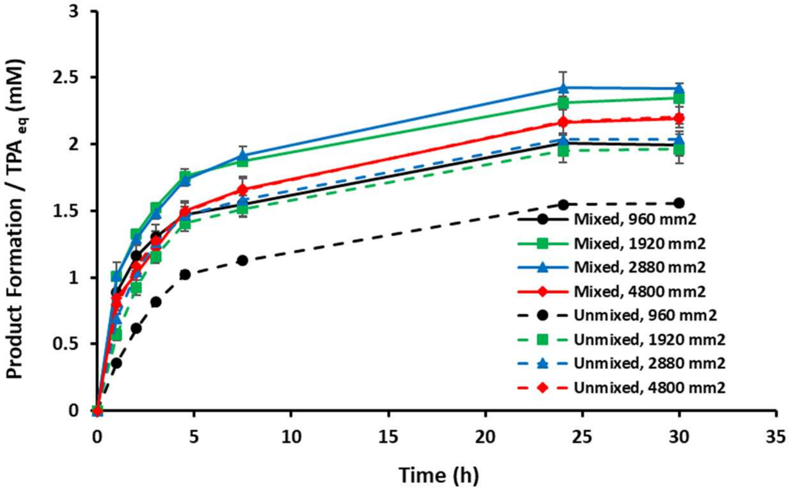

Figure S6. Impact of varying PET surface area on PET degradation (200 nM FAST PETase, 1000 rpm, pH 8, 45°C, 1.5 mL solution in 1.5 mL SealRite tubes), (A) varying the number of amorphous (X_c = 2%) PET films (1 film (~10mg) equate to specific surface area of 6381 mm^2/ g), (B) varying the amount of PET microparticles (X_c = 37.7%) (~10 mg equates to specific surface area of 3.275 mm^2/ g). Error bars indicate the standard deviation of experiments performed in triplicate.

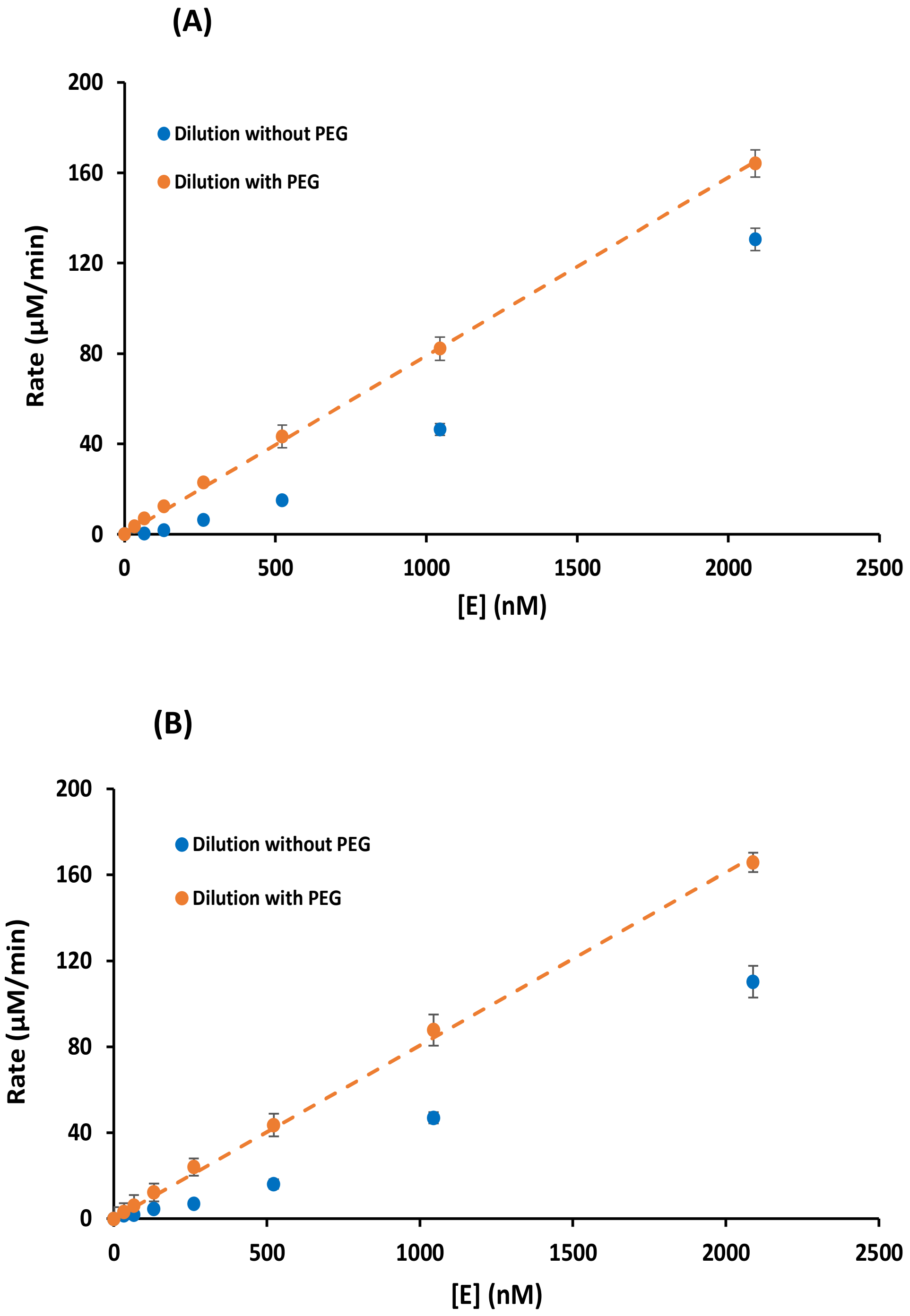

Figure S7. Impact of PEG on linearity of pNA hydrolysis rate vs enzyme concentration, (A) FAST-PETase and (B) HotPETase. The addition of PEG reduces protein loss to tubes and pipette tips. Orange lines represent enzyme samples diluted with PEG containing buffer while blue points represent enzyme samples diluted in buffer without PEG. Error bars indicate the standard deviation of experiments performed in triplicate.

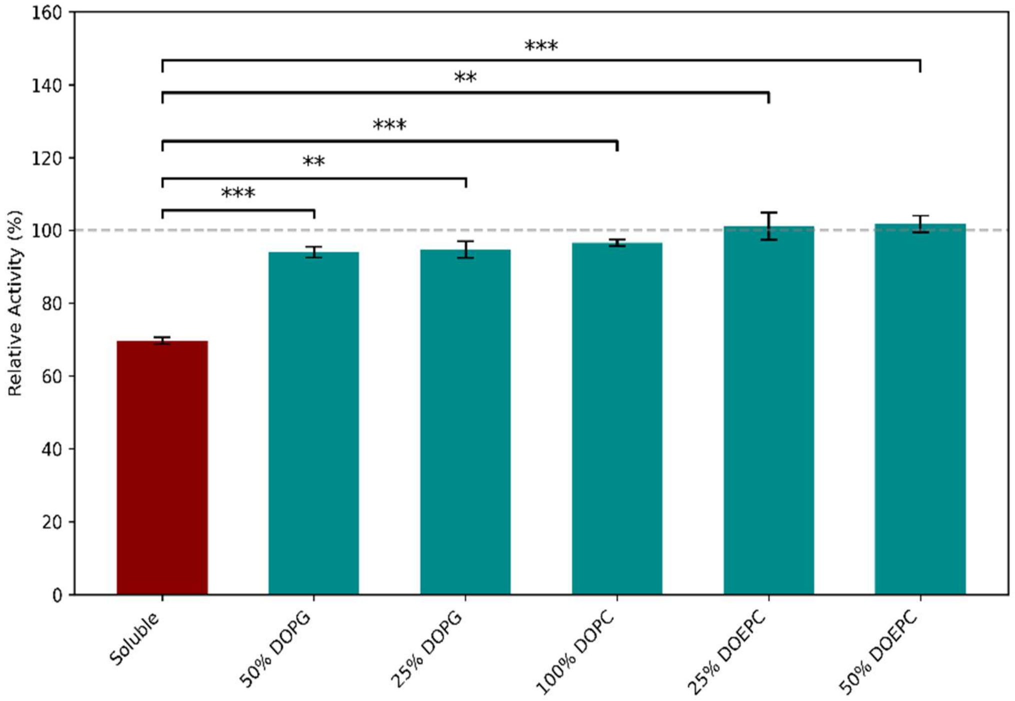

Figure S8. FAST PETase activity retention when subjected to mixing at 2000 rpm for 2 hours at room temperature. Enzymes were tethered to their respective SUVs prior to agitation. **$p < 0.01$, ***$p < 0.001$.

(A)

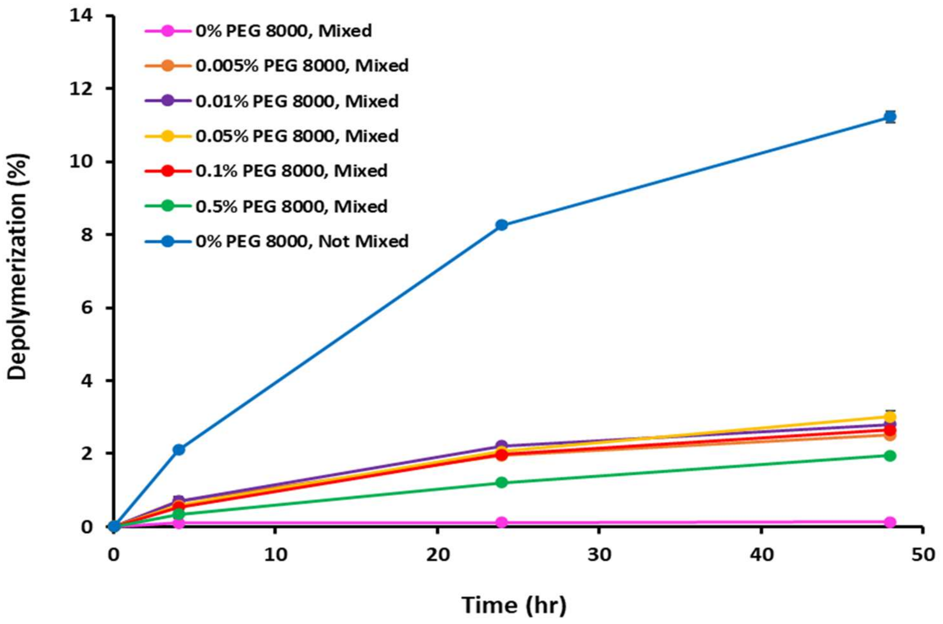

(B)

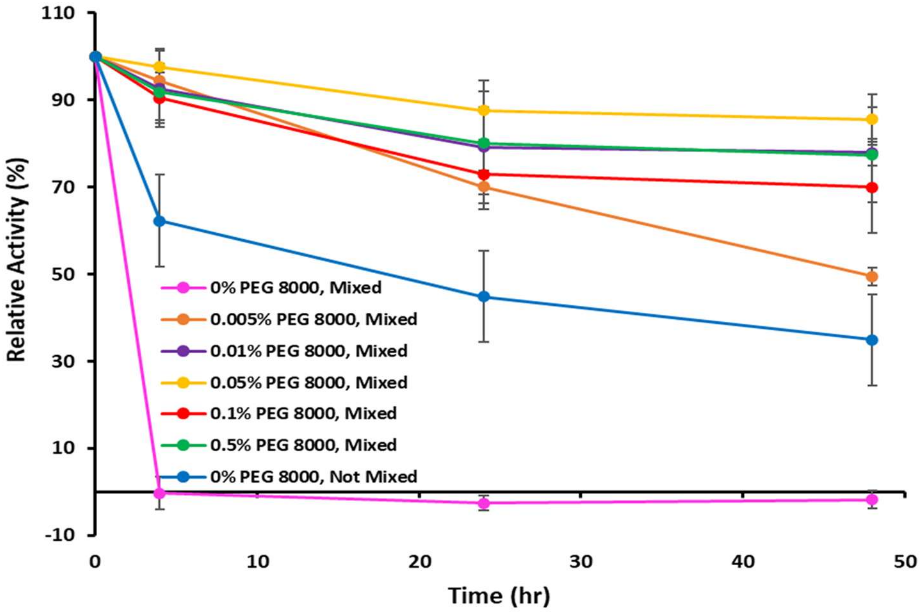

Figure S9. PET degradation and FAST PETase activity with varying concentration of surfactants, (A) PET degradation efficiency in the presence of PEG 8000, (B) relative activity in the presence of PEG 8000. Error bars indicate the standard deviation of experiments performed in triplicate.

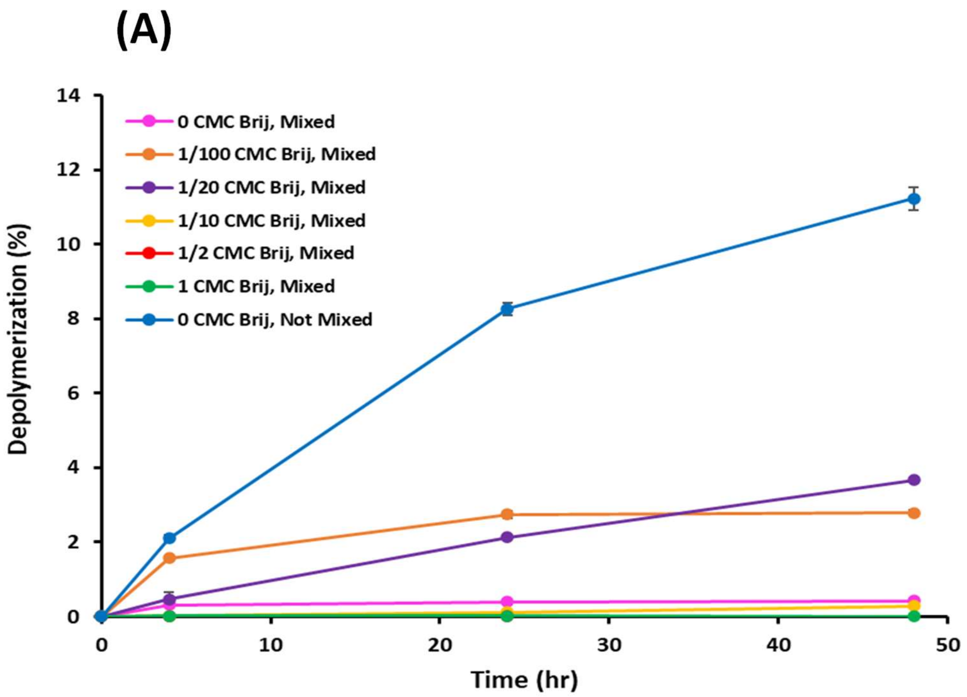

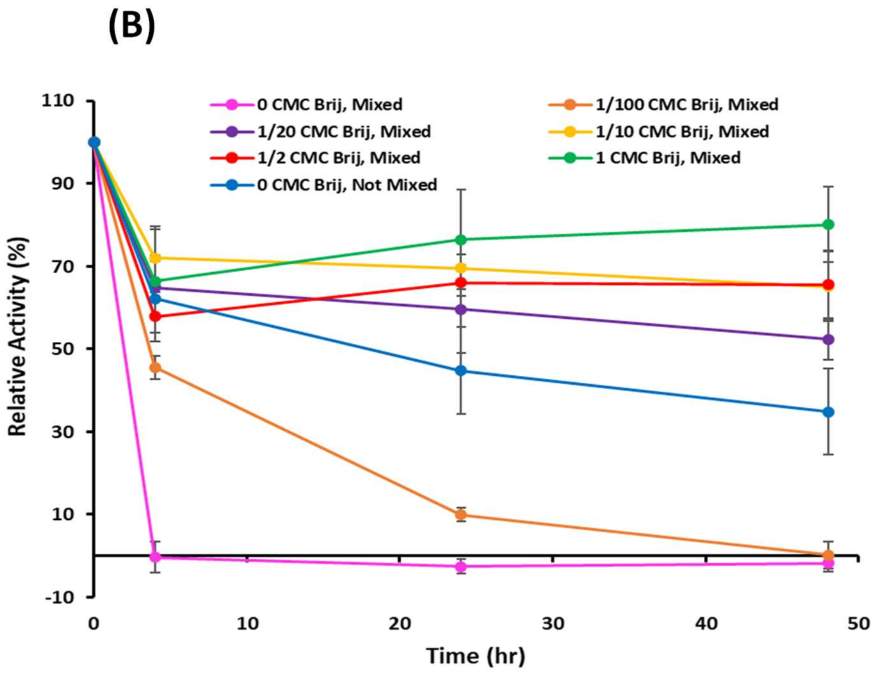

Figure S10. PET degradation and FAST PETase activity with varying concentration of surfactants, (A) PET degradation efficiency in the presence of Brij 35, (B) relative activity in the presence of Brij 35 (200 nM FAST, 2000 rpm mixed, 45°C). Error bars indicate the standard deviation of experiments performed in triplicate.

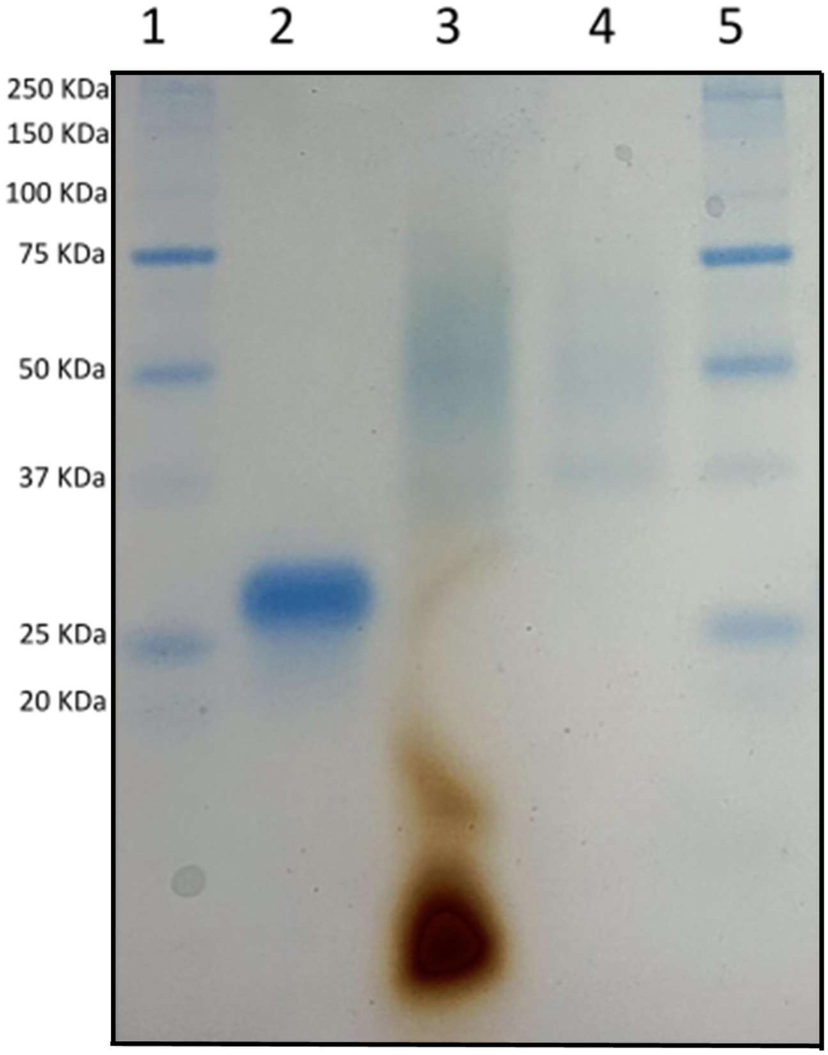

Figure S11. SDS-PAGE and iodine stain gel of FAST-PETase after PEGylation. Lanes (1) BIORAD ladder, (2) unmodified FAST-PETase, (3) 1:25 NHS/ NH_2 molar ratio before purification and stained with iodine, (4) 1:25 NHS/ NH_2 ratio after purification.

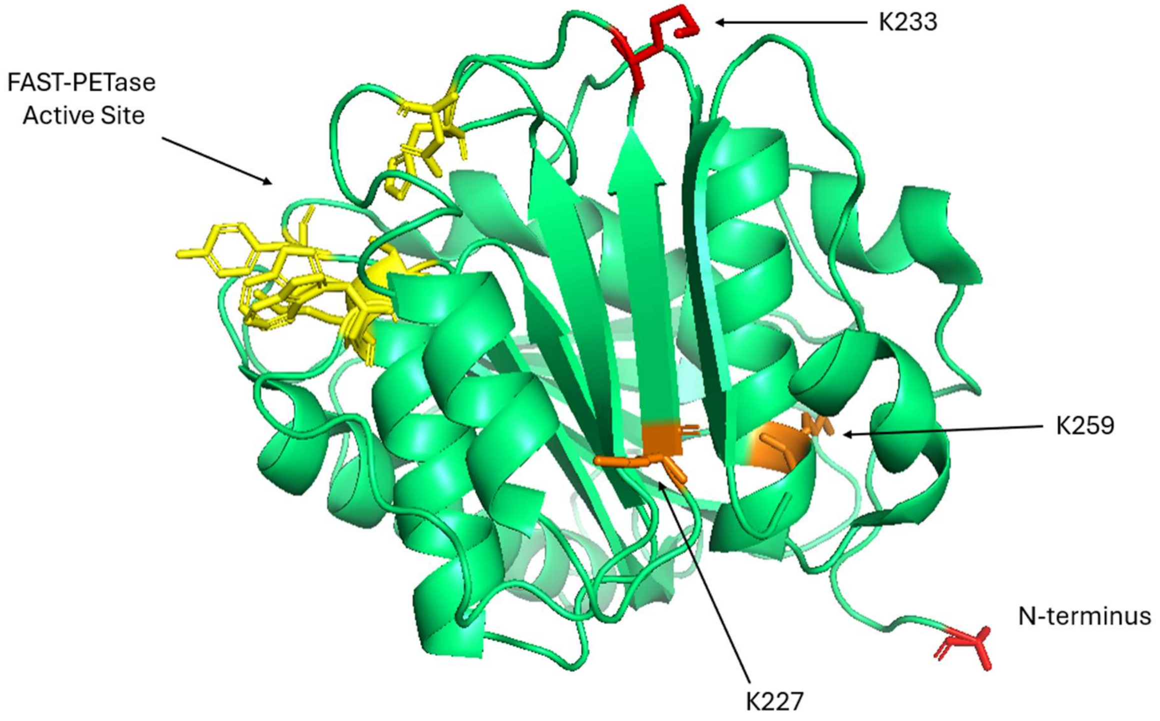

Figure S12. Structure of FAST-PETase (PDB ID 7SH6). The catalytic triad is shown as sticks in yellow. The reactive lysine residues predicted by PRELYM are shown as sticks with those identified to be fast reacting colored red and those identified as slow reacting colored orange.
